# Transcriptomic and Epigenomic Dynamics of Honey Bees in Response to Lethal Viral Infection

**DOI:** 10.3389/fgene.2020.566320

**Published:** 2020-09-24

**Authors:** Hongmei Li-Byarlay, Humberto Boncristiani, Gary Howell, Jake Herman, Lindsay Clark, Micheline K. Strand, David Tarpy, Olav Rueppell

**Affiliations:** ^1^Department of Entomology and Plant Pathology, North Carolina State University, Raleigh, NC, United States; ^2^Department of Biology, University of North Carolina at Greensboro, Greensboro, NC, United States; ^3^High Performance Cluster, Office of Information Technology, North Carolina State University, Raleigh, NC, United States; ^4^High Performance Computing in Biology, University of Illinois at Urbana-Champaign, Urbana, IL, United States; ^5^Army Research Office, Army Research Laboratory, Research Triangle Park, NC, United States; ^6^W.M. Keck Center for Behavioral Biology, North Carolina State University, Raleigh, NC, United States

**Keywords:** alternative splicing, transcriptome, DNA methylation, immune genes, pupa, IAPV, gene expression, comparative genomics

## Abstract

Honey bees (*Apis mellifera* L.) suffer from many brood pathogens, including viruses. Despite considerable research, the molecular responses and dynamics of honey bee pupae to viral pathogens remain poorly understood. Israeli Acute Paralysis Virus (IAPV) is emerging as a model virus since its association with severe colony losses. Using worker pupae, we studied the transcriptomic and methylomic consequences of IAPV infection over three distinct time points after inoculation. Contrasts of gene expression and 5 mC DNA methylation profiles between IAPV-infected and control individuals at these time points – corresponding to the pre-replicative (5 h), replicative (20 h), and terminal (48 h) phase of infection – indicate that profound immune responses and distinct manipulation of host molecular processes accompany the lethal progression of this virus. We identify the temporal dynamics of the transcriptomic response to with more genes differentially expressed in the replicative and terminal phases than in the pre-replicative phase. However, the number of differentially methylated regions decreased dramatically from the pre-replicative to the replicative and terminal phase. Several cellular pathways experienced hyper- and hypo-methylation in the pre-replicative phase and later dramatically increased in gene expression at the terminal phase, including the MAPK, Jak-STAT, Hippo, mTOR, TGF-beta signaling pathways, ubiquitin mediated proteolysis, and spliceosome. These affected biological functions suggest that adaptive host responses to combat the virus are mixed with viral manipulations of the host to increase its own reproduction, all of which are involved in anti-viral immune response, cell growth, and proliferation. Comparative genomic analyses with other studies of viral infections of honey bees and fruit flies indicated that similar immune pathways are shared. Our results further suggest that dynamic DNA methylation responds to viral infections quickly, regulating subsequent gene activities. Our study provides new insights of molecular mechanisms involved in epigenetic that can serve as foundation for the long-term goal to develop anti-viral strategies for honey bees, the most important commercial pollinator.

## Author Summary

Honey bees, the most important managed pollinators, are experiencing unsustainable mortality. Israeli Acute Paralysis Virus (IAPV) causes economically important disease in honey bees, and it is emerging as a model system to study viral pathogen-host interactions in pollinators. The pupation stage is important for bee development but individuals are particularly vulnerable for parasitic mite infestations and viral infections. Currently, it is unclear how honey bee pupae respond to this virus. However, these responses, including gene expression and DNA methylomic changes, are critical to understand so that anti-viral genes can be identified and new anti-viral strategies be developed. Here, we use next-generation sequencing tools to reveal the dynamic changes of gene expression and DNA methylation as pupae succumb to IAPV infections after 5, 20, and 48 h. We found that IAPV causes changes in regions of DNA methylation more at the beginning of infection than later. The activity of several common insect immune pathways are affected by the IAPV infections, as are some other fundamental biological processes. Expression of critical enzymes in DNA methylation are also induced by IAPV in a temporal manner. By comparing our results to other virus studies of honey bees and fruit flies, we identified common anti-viral immune responses. Thus, our study provides new insight on the genome responses of honey bees over the course of a fatal virus infection with theoretical and practical implications.

## Introduction

Infectious disease results from the breakdown of an organism’s normal physiological state because of the presence or proliferation of a pathogen. This disruption can be molecularly characterized by transcriptome profiling to permit a systemic understanding of host-pathogen interactions, particularly in combination with other system-level approaches (Arvey et al., 2012; Tanca et al., 2013; Gardy and Loman, 2018). Transcriptomic and epigenomic changes in response to pathogen infections are important to understand because they are interdependent but relate to different temporal dynamics.

DNA methylation of Cytosine (5 mC) is a central epigenetic regulator of gene expression and alternative splicing (Zemach et al., 2010; Li-Byarlay et al., 2013), affecting diverse aspects of organismal function and disease (Edwards and Myers, 2007). Epigenetic programming may also be the target of host manipulations by pathogens (De Monerri and Kim, 2014) and affect host defenses and susceptibility to infection (Merkling et al., 2015; Kuss-Duerkop et al., 2018). The virus induced changes in host immune response and gene expression via DNA methylation is still a new study field. It is understudied whether changing host DNA methylation such as gene expression of certain antiviral immune genes is a main mechanism utilized by DNA or RNA viruses to manipulate immune responses. Genome-wide analyses of methylome and transcriptome indicated that the infection of DNA tumor virus induced hypermethylation of host genes during viral persistence (Kuss-Duerkop et al., 2018). In plants, DNA hypomethylation was reported after 24 h of viral infection (Pavet et al., 2006).

Honey bees (*Apis mellifera* L.) were the first insects in which a fully functional 5 mC DNA methylation system was discovered (Wang et al., 2006; Kucharski et al., 2008). Social hymenopterans, such as bees, wasps, and ants, have low levels of DNA methylation (Hunt et al., 2010; Glastad et al., 2015; Bewick et al., 2017). The methylated 5 mC sites can be one of three different kinds (CpG dinucleotides, CHG, and CHH, H represents A, T or C) (Lyko et al., 2010). The CpG is the most common kind in honey bees. CpG DNA methylation in insects occurs predominantly in gene bodies, associated with histone modifications and alternative splicing (Lyko et al., 2010; Zemach et al., 2010; Hunt et al., 2013; Li-Byarlay et al., 2013; Glastad et al., 2014; Yan et al., 2014; Glastad et al., 2016). Its phenotypic consequences range from behavioral plasticity (Herb et al., 2012; Opachaloemphan et al., 2018) to alternative development trajectories (Kucharski et al., 2008; Glastad et al., 2019) and disease responses (Galbraith et al., 2015). Especially the study from Herb et al. (2012) showed gene expression differences related to differentially methylated regions (DMRs) and also suggests wider effect because many DMRs are related to transcription factors, in which the alternative splicing may affect their function. Despite this breath of effects, few studies have investigated the role of CpG DNA methylation in insect development, immune system, and disease (Mukherjee et al., 2015; Burggren, 2017; Vilcinskas, 2017; Grozinger and Flenniken, 2019).

Honey bees are the most important managed pollinator in agriculture and crop production (Calderone, 2012). However, recent declines in honey bee health have led to unsustainably high annual colony losses in the apicultural industry (Calderone, 2012; Kulhanek et al., 2017) for which multiple, potentially interacting factors are likely the cause (Cornman et al., 2012; Goulson et al., 2015). One major contributor to widespread colony mortality is pathogenic viral infection. Many of the most important viruses are either associated with or can be directly vectored by parasitic *Varroa* mites (Bailey et al., 1963; Chen and Siede, 2007; Di Prisco et al., 2011; Gisder and Genersch, 2017). Pathogenic viral stressors affect the morphology, physiology, behavior, and growth of honey bees at different developmental stages (Chen and Siede, 2007; Runckel et al., 2011; Cornman et al., 2012; McMenamin and Genersch, 2015), ultimately leading to reduced colony productivity and mortality. The developmental pupal stage is critical for many viral diseases because the *Varroa* mite feeds on the pupae for a prolonged time (Di Prisco et al., 2011).

Honey bees employ a combination of individual- and group-level defenses against pathogens (Wilson-Rich et al., 2009). The main innate immune pathways of insects are present in honey bees and respond to virus infections (Evans et al., 2006; Brutscher et al., 2015), including the Toll, Janus kinase and Signal Transducer and Activator of Transcription (JAK-STAT), Immune Deficiency (Imd), c-Jun NH2-Terminal Kinase (JNK) pathways, antimicrobial peptides (AMPs), endocytosis, and phagocytosis mechanisms (Brutscher et al., 2015). Activation of some immune pathways, such as the RNAi mechanism, can lead to increased virus resistance (Brutscher et al., 2017). Thus, we understand that some honey bee genes respond to virus infection but a systematic characterization of the temporal dynamics of the immune responses and the general transcriptome and methylome have not been investigated, although such studies can yield important information (Lemaitre and Hoffmann, 2007).

Israeli Acute Paralysis Virus (IAPV) has been associated with colony losses (Cox-Foster et al., 2007; Maori et al., 2009; Tantillo et al., 2015), particularly during the winter (Chen et al., 2014). IAPV belongs to the picornavirus-like family Dicistroviridae, that includes single-strand, positive sense RNA viruses that are pathogenic to a range of insects (Bonning and Miller, 2010). IAPV is relatively common in honey bees and affects all life history stages and castes (Chen et al., 2014). When cells are infected, the positive strand of RNA is released into the cytosol and translated by the host ribosomes. Then the assembled complexes for viral replication synthesize negative-sense RNA from which more descendant positive strands of the RNA are produced. These are assembled with viral proteins into virions that are then released to infect other cells (Nagy and Pogany, 2012; Chen et al., 2014). Covert infections persist through vertical and horizontal transmission, but IAPV can also readily cause paralysis and death of infected individuals (Maori et al., 2007). Acute infections of IAPV profoundly affect cellular transcription at the pupal stage (Boncristiani et al., 2013), and microarray analyses of larvae and adults indicate significant and stage-specific responses to IAPV infection (Chen et al., 2014). Parallel transcriptomic and epigenomic profiling of adult IAPV infection identified some additional molecular responses, including expression changes in immune genes and epigenetic pathways (Galbraith et al., 2015). However, little overlap of identified responses within and among studies leave the molecular characterization of IAPV infection in honey bees unresolved, and more detailed studies of dynamic responses in the time-course of IAPV infection are needed.

Here, we experimentally test the hypothesis that viral infections can affect not only transcriptomic profiles but also induce DNA methylomic changes in honey bee pupae. Specifically, we compared the complete transcriptome and methylome profiles across three distinct phases of a lethal IAPV infection. Furthermore, we tested if our results indicate significant overlap of immune genes with fruit flies as well as other stages of honey bees under IAPV viral infections.

## Results

### Temporal Viral Infections Cause Transcriptomic Changes in Gene Expressions

**FIGURE 1:**
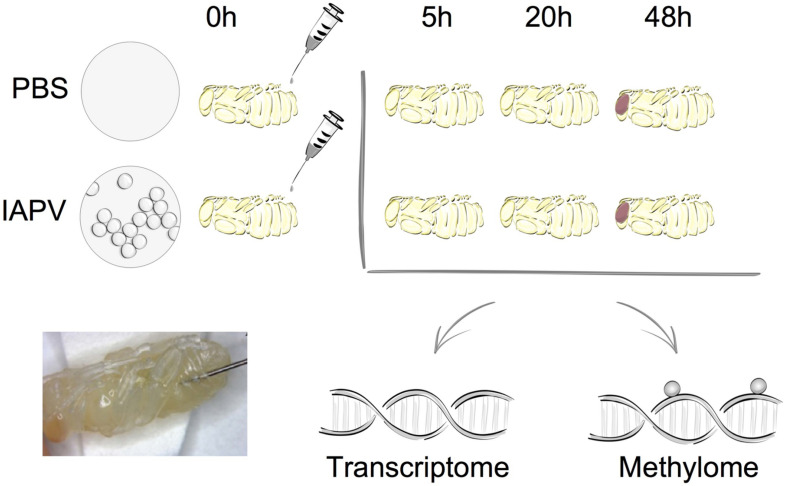
Overall experimental design. The two experimental groups included either sham treatment (PBS injection) or Israeli Acute Paralysis Virus (IAPV) inoculations via injection. At each investigated time point (5, 20, or 48 h post-injection) three biological replicates were collected, each consisting of one whole pupa that was individually processed for further transcriptomic and methylomic analyses.

The transcriptomes of whole honey bee pupae injected with PBS or infected with IAPV were compared at three time points: 5, 20, and 48 h post-injection (Figure 1). On average, IAPV-infected pupae yielded slightly more RNA-seq reads than the corresponding PBS controls (5 h: IAPV = 46,001,777 vs. PBS = 41,666,177; 20 h: IAPV = 47,915,086 vs. PBS = 42,429,737; and 48 h: IAPV = 42,649,987 vs. PBS = 39,248,297). Coverage thus varied among individual samples from 41x to 65x (Supplementary Table 1). Differentially expressed genes (DEGs) increased with disease progression over time from 432 after 5 h to 5,913 after 20 h and 5,984 after 48 h (Supplementary Table 2).

**FIGURE 2:**
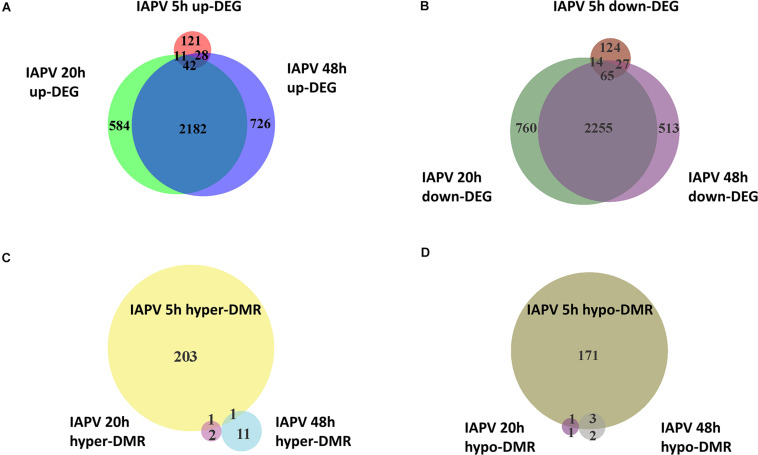
Directional overlap of differentially regulated genes among time points. **(A)** Venn diagram showing genes that were differentially expressed as up-regulated (FDR < 0.05). Number of DEGs only in post IAPV 5, 20, and 48 h are 121, 584, and 726. Number of genes between IAPV 5 and 20 h only overlap is 11. Number of DEGs between IAPV 5 and 48 h only overlap is 28. Number of DEGs between IAPV 20 and 48 h only overlap is 2182. Number of DEGs from IAPV 5–20–48 h overlap is 42. **(B)** Venn diagram showing genes that are differentially expressed as down-regulated (FDR < 0.05). Number of DEGs only in post IAPV 5, 20, and 48 h are 124, 760, and 513. Number of DEGs between IAPV 5 and 20 h only overlap is 14. Number of DEGs between IAPV 5 and 48 h only overlap is 27. Number of DEGs between IAPV 20 and 48 h only overlap is 2255. Number of DEGs from IAPV 5–20–48 h overlap is 65. **(C)** Venn diagram showing genes that are differentially methylated (percent methylation difference larger than 10%, *q* < 0.01) as hyper-methylation among three time points (5, 20, and 48 h after IAPV infection). Number of DMRs only in post IAPV 5, 20, and 48 h are 203, 2, and 11. Number of DMRs between IAPV 5 and 20 h only overlap is 1. Number of DMRs between IAPV 5 and 48 h only overlap is 1. **(D)** Venn diagram showing genes that are differentially methylated (percent methylation difference larger than 10%, *q* < 0.01) as hypo-methylation among three time points (5, 20, and 48 h after IAPV infection). Number of DMRs only in post IAPV 5, 20, and 48 h are 171, 1, and 2. Number of DMRs between IAPV 5 and 20 h only overlap is 1. Number of DMRs between IAPV 5 and 48 h only overlap is 3.

Relative to the PBS control, IAPV-infected pupae had 198 genes significantly up-regulated and 234 genes significantly down-regulated after 5h; 2,738 genes were significantly up-regulated and 3,175 genes significantly down-regulated after 20 h; and 3,021 genes significantly up-regulated and 2,963 genes significantly down-regulated after 48 h (Supplementary Figures 1–3). DEG overlap between all significantly up- or down-regulated genes was significant in all pairwise directional comparisons (up- and down-regulated genes separately) among the three time points (Figure 2; up-regulated 5 h vs. 20 h: *p* < 0.05; all other tests: *p* < 0.001). For the > 8-fold DEGs, significant overlap was found for all pairwise comparisons among the IAPV up-regulated gene sets (*p* < 0.001) and also for the down-regulated gene sets at 20 and 48 h (*p* < 0.001) (Supplementary Table 2).

### Gene Enrichment and Functional Analyses

**FIGURE 3:**
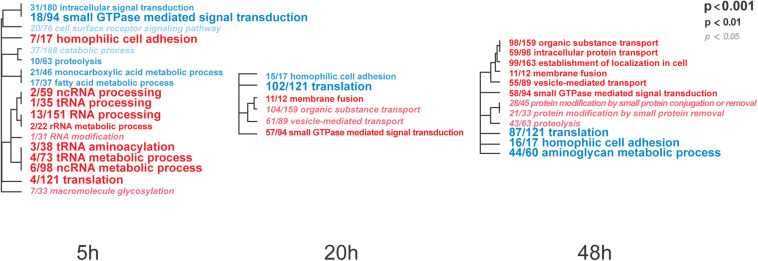
Overall trends in biological processes affected by progressing IAPV infection. Gene Ontology Mann-Whitney U tests of the expression differences between IAPV and control samples at the three time points included all data points regardless of their individual level of statistical significance. Red and blue fonts indicate GO terms that are enriched in up- and down-regulated DEGs in IAPV relative to control samples, respectively. Font size indicates the level of significance for the particular GO term and clustering is based on empirical GO-term similarity in our dataset.

To identify IAPV-induced expression patterns across all genes regardless of significance threshold, GO Mann-Whitney *U*-tests and a Weighted Gene Co-expression Network Analysis were performed on the expression differences between IAPV infected and control samples at three time points. Post 5h of IAPV infection, multiple biological process GO terms were observed with upregulation of RNA processing (ncRNA, tRNA, and rRNA) as the most significant GO terms (*p* < 0.001). Only one term was significantly downregulated at this time point: “small GTPase mediated signal transduction.” 20 h post IAPV infection, no GO terms passed the strictest significance threshold (*p* < 0.001) for upregulation, and GO term “translation” was the most significantly downregulated (*p* < 0.001). Finally, in post 48h IAPV infected samples, there was still no term passing the strictest significance in upregulation, and GO terms of “translation,” “homophilic cell adhesion,” and “aminoglycan metabolic process” were the most significantly downregulated (Figure 3). As to molecular function, significant GO terms associated with downregulation in post 20 and 48 h IAPV infected samples were “structural molecules,” “structural constituent of ribosome,” “structural constituent of cuticle,” and “chitin binding” (Supplementary Figure 4). As to cellular component, significant GO terms associated with downregulation in post 20 and 48 h IAPV infected samples were “ribosomal subunit,” “ribonucleoprotein complex,” and “intracellular non-membrane organelle” (Supplementary Figure 4). Although numerous other terms were less significantly enriched in up- or down-regulated genes, no other GO terms surpassed the most stringent significance criterion.

### Viral and Ribosomal RNA Content

Four transcripts (IAPV, Deformed Wing Virus (DWV), Large Sub-Unit rRNA (LSU rRNA), and Small Sub-Unit rRNA (SSU-rRNA) accounted for 63.5% of all sequenced reads (Supplementary Figure 5). As expected, a large number of IAPV reads was observed in the IAPV-infected pupae, with the proportion being similarly high at 20 h (86% ave) and 48 h (90% ave). Additionally, five of nine control samples (all time points included) had a large proportion of reads mapping to DWV.

### Comparative Transcriptomic Analyses of Immune Genes With Other Pathogen Infection in Honey Bees and *Drosophila*

**FIGURE 4:**
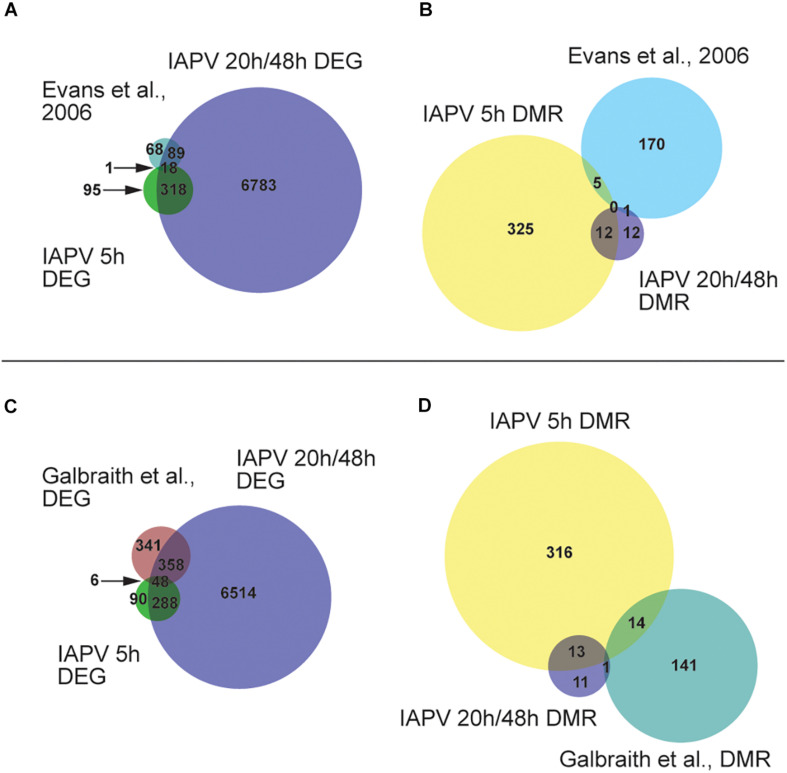
Comparisons or our results to other studies revealed significant overlap. **(A)** Venn diagram showing overlap of genes that are differentially expressed (FDR < 0.05; *p* < 0.05). Number of DEGs (differentially expressed genes) only in IAPV 5, 20/48 h, or Evans et al. (2006) immune gene list is 95, 6783, or 68. Number of DEGs overlap between IAPV 20 h/48 h and Evans et al. is 89. Number of DEGs overlap between IAPV 5 and 20 h/48 h is 318. Number of DEGs overlap between IAPV 5 h and Evans et al. is 1. **(B)** differentially methylated (>10%, *p* < 0.05) among three time points (5, 20, and 48 h after viral infection) and immune gene list from Gisder and Genersch (2017). Number of DMRs (differentially methylated regions) only in IAPV 5, 20/48 h, or Evans et al. (2006) immune gene list is 325, 12, or 170. Number of DMRs overlap between IAPV 20 h/48 h and Evans et al. is 1. Number of DMRs overlap between IAPV 5 and 20/48 h is 12. Number of DMRs overlap between IAPV 5 h and Evans et al. is 5. **(C)** Venn diagram showing overlap of DEGs (FDR < 0.05; *p* < 0.05) compared to Galbraith et al. Number of DEGs only in IAPV 5, 20/48 h, or Galbraith et al. is 90, 6514, or 341. Number of DEGs overlap between IAPV 20 h/48 h and Galbraith et al. is 358. Number of DEGs overlap between IAPV 5 and 20 h/48 h is 288. Number of DEGs overlap between IAPV 5 h and Galbraith et al. is 6. **(D)** differentially methylated (>10%, *p* < 0.05) among three time points (5, 20, and 48 h after IAPV infection) and immune gene list from Galbraith et al. DMR list. Number of DMRs (differentially methylated regions) only in IAPV 5, 20 h/48 h, or Galbraith et al. DMR list is 316, 11, or 141. Number of DMRs overlap between IAPV 20 h/48 h and Galbraith et al. is 1. Number of DMRs overlap between IAPV 5 and 20 h/48 h is 14. Number of DMRs overlap between IAPV 5 h and Galbraith et al. is 13.

A comparison with a previously published list of 186 immune genes from Evans et al. (2006) showed that 1 and 89 of them were represented in our 5 h, 20 h/48 h DEG lists respectively (Figure 4A and Supplementary Tables 3, 4). The overlap between immune genes and our DEGs was significant for up-regulated DEGs at 5 h and down-regulated DEGs at 20 h (Supplementary Tables 3, 4). Comparison of our data with IAPV-induced gene expression changes in adult honey bees in Galbraith et al. (2015) revealed 6 and 358 genes overlapped, indicating a significant directional overlap of our up- and down-regulated DEGs at 5 h and 20 h/48 h with DEGs in adults (Figure 4C, for specific gene names, see Supplementary Tables 3, 4). When compared to immune genes associated with anti-viral responses in *Drosophila* (Xu et al., 2012), 11, 212, and 219 genes overlapped in our 5h, 20h, and 48h DEG lists (Supplementary Table 5), representing significant directional overlap of our up- and down-regulated DEGs at 20 h and 48 h (for specific gene names see Supplementary Table 5).

### Temporal Dynamics of Genes and Molecular Pathways Based on Transcriptomic Data

Analyses of the three infection time points revealed dramatic changes of gene expression levels from several key metabolic and immune pathways, including lipid metabolism, the Jak-STAT pathway, and AMPs. With regards to lipid metabolism, the expression of the gene *Cyp6as5* was significantly increased in the infected samples from 5 to 20 h, and from 20 to 48 h, similarly to 9 more genes that displayed similar expression profiles (Supplementary Figure 7).

In the Jak-STAT immune pathway, the expression of gene *SoS* was significantly increased in the infected samples from 20 to 48 h (Supplementary Figure 6). Nine more genes displaying similar expression profiles as *SoS* after viral infection were identified (Supplementary Figure 7). Among the AMP genes, the expression of gene *defensin-1* was significantly increased in the infected samples from 5 to 20 h, and from 20 to 48 h (Supplementary Figure 6). The gene expression of EGFR and a group of other genes were transiently downregulated from 5 to 20 h post-infection and from 20 to 48 h post-infection by IAPV (Supplementary Figure 7). Gene *GB55029* (uncharacterized) showed an increase from 5 to 20 h infection, but then a significant decrease of expression from 20 to 48 h infection (Supplementary Figure 7).

### Differentially Spliced Genes

**FIGURE 5:**
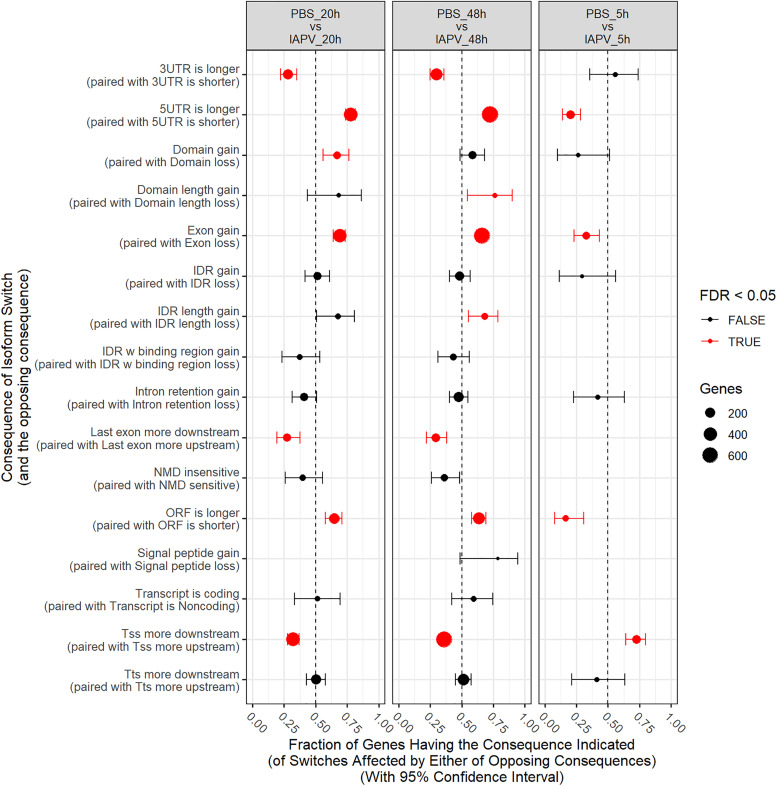
Significant trends in consequences of isoform switches or alternative splicing were observed, and were generally similar at 20 and 48 h, often with opposite consequences at 5 h. Fraction of genes having the consequence indicated with 95% Confidence Interval. UTR-untranslated region, ITR-Inverted Terminal Repeat, ORF-open reading frame, NMD- nonsense-mediated decay, TSS-transcription start site, TTS-transcription terminal site.

Number of genes with significant isoform switches in samples 5, 20, and 48 h post IAPV infection were 193, 620, and 990 (Supplementary Figure 8 and Supplementary Table 6). Number of significant differential transcripts usage (DTU) or alternative splicing is much higher post 20 and 48 h of IAPV infection (Supplementary Figure 9). All the splicing events are categorized in Table 1. Among all the categories, there are significant trends of DTU to be in shorter 3′ UTRs, longer 5′ UTRs, Exon gain, Transcription Start Site (TSS) more downstream in 20 and 48 h IAPV post infection treatments (Figure 5).

**TABLE 1:** Isoform switch consequences investigated.

Category	Consequence	Description	Post 5 h	Post 20 h	Post 48 h
1	Tss	Change in transcription start site	120	407	602
2	Tts	Change in transcription termination site	21	169	246
3	Last exon	Last exon changed	9	97	130
4	Intron retention	Difference in intron retention	26	100	186
5	Intron structure	Different exon-exon junctions used	160	552	875
6	Exon number	Different number of exons	86	373	627
7	ORF seq similarity	Jaccard similarity of AA sequences below 0.9	47	211	281
8	ORF genomic	Change in genomic position of ORF	97	382	540
9	5 utr length	Difference in length of 5′ UTR	131	433	653
10	3 utr length	Difference in length of 3′ UTR	27	190	284
11	NMD status	Change in sensitivity to nonsense-mediated decay	5	45	76
12	Coding potential	Change in coding potential probability, above or below 0.7	5	30	36
13	Domains identified	Change in which protein domains were identified	19	96	119
14	Domain length	Change in length of overlapping domains	3	20	26
15	IDR identified	Difference in presence of intrinsically disordered regions	19	112	159
16	IDR length	Difference in length of intrinsically disordered regions	5	38	45
17	IDR type	Difference in presence of binding site in IDR	7	42	63
18	Signal peptide identified	Change in presence of signal peptide	1	8	14

*Numbers of significant genes are shown.*

### Enzymes Involved in DNA Methylation

**FIGURE 6:**
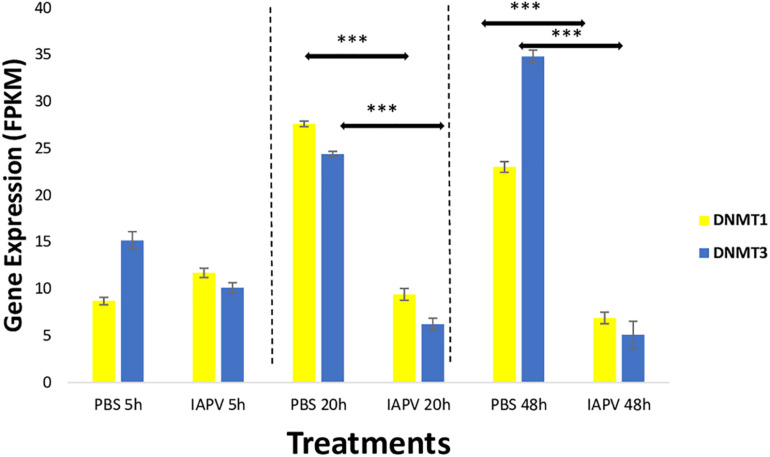
Gene expression patterns (mean ± SE) of the major DNA Methyl-Transferases (DNMTs). Both DNMT1 (GB47348) and DNMT3 (GB55485) showed significant down-regulation when comparing sham control (*N* = 3) and IAPV samples (*N* = 3) after 20 and 48 h after IAPV infection respectively. ^∗∗∗^*p* < 0.001. False Discovery Rate (FDR) < 0.05.

Gene expression differences were also identified in the DNA methylation system, specifically the two important enzymes, *DNA methyl-transferase 1* (*DNMT1*) and *3* (*DNMT3*). When compared between IAPV and control samples, *DNMT1* (*GB47348*) was significantly down-regulated 1.5-fold (*p* < 0.001) at 20 h post-infection, and 1.7-fold (*p* < 0.001) at 48 h post-infection (Figure 6). Similarly, *DNMT3* (*GB55485*) was significantly down-regulated 1.7-fold (*p* < 0.001) in 20 h post-infection samples, and 2.7-fold (*p* < 0.001) in 48 h post-infection samples (Figure 6). There was no significant difference of gene expression between IAPV and controls post 5 h post-viral infection (Figure 6).

### Changes of DNA Methylation After Viral Infections and Comparative Methylomic Analysis

**FIGURE 7:**
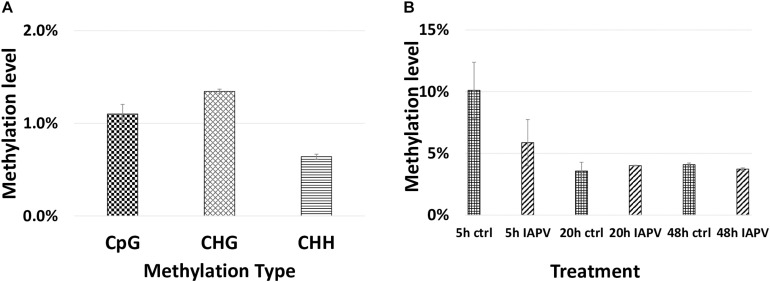
Genome wide methylation patterns. **(A)** Total methylated 5 mC sites in each group (CpG, CHG, and CHH) are shown. **(B)** Methylated CpG sites/Total CpG sites are shown for each treatment and time point.

Total methylated 5 mC sites in CpG, CHG, and CHH settings were 1,955,471; 878,838; and 1,433,905 respectively (Figure 7). The ratio of methylated CpG sites versus total CpG sites was highest in 5 h PBS control (10%), followed by 5 h IAPV post-infection samples, 20 h PBS control, 20 h IAPV post-infection samples, 48 h control, and lowest in 48 h IAPV post-infection samples (about 4%) (Figure 7). The whole genome-wide Pearson correlation matrix for CpG base profiles across all samples at each time point (5, 20, and 48 h post-infection) are listed in Supplementary Figure 10.

We identified 523, 5, and 18 differentially methylated regions (DMRs) between IAPV and control groups in the 5, 20, and 48 h post-infection samples, respectively (Supplementary Table 7 for a detailed gene list). Significant overlap of DMRs existed between 5 and 20 h (*p* < 0.05) and 5 and 48 h (*p* < 0.0001) comparisons (Supplementary Table 4).

Our comparison of our DMR lists with the previously published immune genes of Evans et al. (2006) revealed 5 and 1 gene were overlapping with the DMRs at 5 h and 20 h/48 h post-infection, respectively (Figure 4B and Supplementary Tables 3, 4). Comparison of our data with IAPV-induced methylation changes in adult honey bees of Galbraith et al. (2015) revealed 14 and 1 genes were overlapped with DMRs at 5 h and 20 h/48 h (Figure 4D, for specific gene names see Supplementary Tables 3, 4).

We examined the genome-wide Pearson correlation matrix for CpG base profiles across all samples at each time point. Overall samples after 5 h infection have the highest correlation (0.72–0.87), and samples after 48 h infection have the lowest correlation (0.69–0.73); specific coefficients are listed in Supplementary Figure 10).

### Differential DNA Methylation Leads to Differential Gene Expression Later in Several Immune Pathways

To look into the potential association between gene expression and CpG methylation, we studied whether genes that showed early changes of CpG methylation also exhibited significant changes of gene expression later. A list of 38 immune genes that were hypo- or hyper-methylated at the first time point (5 h post-IAPV infection), were found differentially expressed later (48 h post IAPV infection) (Supplementary Table 8). These cellular pathways included the MAPK (mitogen-activated protein kinase) pathway, JAK/STAT (Janus kinase/signal transducer and activator of transcription) pathway, Hippo signaling pathway, mTOR (mammalian target of rapamycin) signaling pathway, TGF-beta (transforming growth factor-beta) signaling pathway, ubiquitin mediated proteolysis, and spliceosome. Interestingly, we detected 12 genes from this group with differentially spliced patterns with a significant statistical q value (Table 2).

**TABLE 2:** A list of twelve immune genes that were hypo- or hyper-methylated first post 5 h of viral infection, then gene expression and splicing patterns changed significantly post 48 h of viral infection (*q* < 0.05).

Gene.function	BEEBASE	X5h.meth.diff	X48h.DEG. log2_fold_change	Gene name	Consequences_5h	Consequences_20h	Consequences_48h
Hippo signaling pathway	GB53036	−40.02	1.63	Serine/threonine-protein kinase Warts, transcript variant X2	NA	NA	5
Lysine degradation	GB47635	−38.66	−0.5	Suppressor of variegation 3–9, transcript variant X1	NA	NA	1,4,6,8,9,17
FoxO signaling pathway	GB54054	−26.65	2.08	Ubiquitin carboxyl-terminal hydrolase 7, transcript variant X1	1,4,5,8,9,15	NA	NA
Spliceosome	GB46655	−16.15	1.78	Splicing factor U2AF 50 kDa subunit, transcript variant X1	NA	5,6,8,9	NA
Lysine degradation	GB43459	24.34	−2.42	Histone-lysine N-methyltransferase trithorax, transcript variant X2	NA	NA	4,6,10
Ubiquitin mediated proteolysis	GB42193	26.51	1.53	Ubiquitin-conjugating enzyme E2-22 kDa, transcript variant X1	NA	1,5,7,8,14	NA
Ubiquitin mediated proteolysis	GB45517	29.47	0.65	E3 ubiquitin-protein ligase Nedd-4, transcript variant X6	NA	NA	2,3,5,6,8,10,17,
TGF-beta signaling pathway	GB43676	30.93	1.37	Activin receptor type-1, transcript variant X1	NA	NA	5,6,8
uncharacterized	GB41710	31.91	−2.07	Uncharacterized LOC408494, transcript variant X3	NA	5,6,7	1,6
mTOR signaling pathway	GB52079	33.8	−0.56	Rapamycin-insensitive companion of mTOR, transcript variant X2	NA	NA	1,5,9
Lysosome	GB54097	40.23	2.52	Malvolio, transcript variant X1	NA	1,5,8,9	1,5,8,9
RNA transport	GB51710	45.25	1	Eukaryotic initiation factor 4A-I, transcript variant X3	NA	NA	2,3,6,10,11

*Gene.function (column 1) showed immune pathways involved. BEEBASE (column 2) showed the gene ID in BEEBASE. X5h.meth.diff (column 3) showed changes of DNA methylation level of the gene/region when comparing post 5h IAPV infection samples and control samples. X48h.DEG.log2_fold_change (column 4) showed fold change of gene expression in log2 scale when comparing post 48h IAPV infection samples and control samples. GENENAME (column 5) showed gene names. Consequences_5h, consequences_20h and consequences_48h (column 6–8) are the detected categories of alternative splicing listed in the first column of Table 1. Details of gene IDs and q-values are in Supplementary Table 8.*

### Comparative Methylomic Analyses of Immune Genes With Other Pathogen Infection

The comparison with a previously published list of 186 immune genes from Evans et al. (2006), showed that 5 (*GB45248*, *GB46478*, *GB55483*, *GB51399*, and *GB41850*) and 1 (*GB46478*) genes overlapped in our 5h and 48h DMR lists, respectively (Supplementary Table 3). There was no significant directional overlap of the list of immune genes and our hyper- or hypo-regulated DMRs at 5, 20, and 48 h (Supplementary Tables 3, 4). Comparison of our data with IAPV-induced gene expression changes in adult honey bees in Galbraith et al. (2015) revealed 19, 1 (*GB51998*), and 1 genes (*GB42022*) overlapped, indicating no significant directional overlap of our hyper- or hypo-regulated DMRs at 5, 20, and 48 h (Supplementary Tables 3, 4). When compared to immune genes associated with anti-viral responses in *Drosophila* (Xu et al., 2012), 20, 1 (*GB17743*), and 1 genes (*GB15242*) overlapped in our 5, 20, and 48 h DMR lists (Supplementary Table 5).

### Motif

To reveal the potential regulation of CpG methylation, we examined the 6-mer motif of each CpG site, including the 2 nucleotides upstream and downstream of each CpG site. Analysis of 6-mer motifs from 5 h DMRs showed no common pattern of the motif (Supplementary Figure 11). Similarly, analysis of motifs from 20 h DMRs containing CpG sites revealed little signal beyond the selected central CpG sites in the middle of 6-mer motifs. However, DMRs from 48 h post- IAPV infection tend to have a T or C following the central CpG (Supplementary Figure 11).

## Discussion

To test our hypothesis that viral infections not only affects gene expression profiles but also DNA methylomic marks in honey bee pupae, we compared the complete transcriptome and methylome responses across three distinct phases (5, 20, and 48 h) of a lethal IAPV infection. While methylation is most affected early during the infection, differential gene expression increases over time and severity of the infection. Many up- and down-regulated genes are involved in RNA processing and translations, proteolysis, and metabolic processes (Figure 3). A collection of genes have differential transcript usage or alternative splicing post IAPV infections (Figure 5). When compared to differentially expressed genes from adult honey bee and fruit fly post viral infection, we have discovered significant overlap of our IAPV-responsive genes with immune genes from these other studies (Evans et al., 2006).

The pupal stage is a critical phase for metamorphosis and an important temporal window for *Varroa* mite parasitism. Our study, which incorporates and confirms the separate observations of adult or larval honey bees (Cox-Foster et al., 2007; Cornman et al., 2013; Chen et al., 2014; Galbraith et al., 2015), includes new observations that provide a more complete understanding of how the pupae respond to lethal viral attack. Previous studies showed that honey bee pupae can respond to disease with increased immune gene expression to decrease the success of parasites and pathogens (Kuster et al., 2014; Brutscher et al., 2017). Our results reveal that rapid epigenetic changes in response to viral infection precede and presumably trigger profound and widespread transcriptome responses in honey bees. A combination of both transcriptomics and methylomics at multiple time points can reveal the role of and the relation between 5 mC methylation and gene expression profiles under viral infection of insects (Huang et al., 2019).

Alternative splicing in response to viral infection can affect subsequent gene expression through several mechanisms (De Maio et al., 2016). Our results of differential transcript usage or alternative splicing indicated the viral infection can cause large changes in alternative splicing of the bee hosts, which brings a new perspective on molecular mechanisms of IAPV virus pathogenesis. It is known that RNA virus can alter host mRNA splicing (Dubois et al., 2014; Chan et al., 2019). Previous research revealed that DNA methylation is linked to regulate alternative splicing in honeybees via RNA interference knocking down DNMT3 (Li-Byarlay et al., 2013).

The epigenetic changes we report here are interesting because the 5 mC methylome profile indicated a large amount of DMRs after 5 h IAPV infection. However, DMR number dramatically dropped after 20 h and 48 h of infection. This phenomenon may be explained by the cell death and apoptosis after severe infections after 20 and 48 h. We propose a possible explanation is that 5 mC changes as a primary host-defense mechanism responding to viral infection at the onset. During the early stage of viral infection, 5 mC methylation may be the first to react leading to molecular changes genome-wide, potentially interacting with the transcription process such as transcription factor binding. The mechanism on how to induce abnormal methylation at the molecular and cellular levels by viral infection is yet to be determined. A few potential explanations are that pathogenic infection may lead to cellular proliferation and inaccuracy in epigenetic processes, or molecular signaling pathways involved in the infection response affect these epigenetic processes. Previous studies showed that viral infection can change 5 mC methylation in human cancer (Kusano et al., 2006; Toyota and Yamamoto, 2011). Other studies also showed that changes in epigenetics marks can affect immune responses, cell-cycle checkpoints, cell death, and cell fate (Toyota and Yamamoto, 2011; Turner and Diaz-Munoz, 2018).

In honey bees, some evidence links 5 mC methylation marks to gene regulation (Kucharski et al., 2008; Li-Byarlay et al., 2013; Wang and Li-Byarlay, 2015; Li-Byarlay, 2016; Glastad et al., 2019) but limited reports showed 5 mC methylation are associated with change of gene expressions (Herb et al., 2012). The data here indicates that 38 of the initially differentially methylated genes subsequently exhibit significant changes of gene expression (Table 1), and 12 of these genes displayed significant changes in their splicing events. In general, the epigenetic control of 5 mC methylation and gene expression has been reported for plants and mammals (Allis and Jenuwein, 2016; Saripalli et al., 2020), but the function of 5 mC methylation in the regulation of genes in insects is still not clear (Li-Byarlay et al., 2013; Wang and Li-Byarlay, 2015). One hypothesis is that DNA methylation is involved in cell signaling or involved in the regulation of co-transcription via transcription factors (Toyota et al., 2009; Shukla et al., 2011; Marina et al., 2016).

DNA methyl-transferases 1 and 3 are critical enzymes for a functional methylation system. Our data show the IAPV infection reduced the gene expression of these two key enzymes over time. This finding is consistent with previous reports showing that viral infection can change the expression of DNMTs in animal and cell studies (Fang et al., 2012; Tian et al., 2013). The significant fold change of these enzymes is moderate but sufficient to cause dramatic changes in the global DNA methylome, as indicated in previous studies (Wang et al., 2006; Kucharski et al., 2008; Li-Byarlay et al., 2013). The DNA methylation changes in turn can have further consequences for widespread gene expression patterns. We found more DMR post 5 h IAPV infection, when the DNMTs are not yet differently expressed. Possible explanation is that the initial viral infection induced the methylation changes at the initial stage by other potential molecular mechanism such as histone modifications (Paschos and Allday, 2010; Dantas Machado et al., 2015). DNMTs may be regulated to restore homeostasis which may explain why fewer DMRs at the later stages (Matilainen et al., 2017).

At every time-point, we identified numerous differentially expressed genes that confirmed the profound consequences of IAPV infection (Boncristiani et al., 2013). Such effects can certainly be caused by cell expansion or loss, rather than direct effects on gene activity. However, the systemic consequences for the health and function of the organism may be similar: the level of immunity might drop due to declining cell numbers or lowered gene activity per cell. Molecular pathways from differentially expressed and methylated genes indicated that the MAPK signaling pathway, Jak/STAT signaling pathway, Hippo signaling pathway, mTOR signaling pathway, TGF-beta signaling pathway, ubiquitin mediated proteolysis, and spliceosome are critical to respond to viral infections. Both MAPK and Jak/STAT signaling pathway are immune pathways with antiviral immune response in honey bees and dipterans (Dostert et al., 2005; Lemaitre and Hoffmann, 2007; Cirimotich et al., 2010; Chen et al., 2014; Galbraith et al., 2015; Brutscher et al., 2015; Grozinger and Flenniken, 2019). The Hippo signaling pathway is highly conserved from insects to mammals, which regulates cell death and differentiation (Munoz-Wolf and Lavelle, 2017). Previous study also showed differentially expressed genes regulated by IAPV in honey bee brood are involved in the mTOR, TGF-beta pathways, and ubiquitin mediated proteolysis (Chen et al., 2014).

In addition, our data showed that IAPV infection can induce *Defensin-1*, a key antimicrobial peptide responding to viral infections (Kuster et al., 2014; Brutscher et al., 2015). P450 genes are known as responding to oxidative stress in *Drosophila* during larval development (Li et al., 2008). In honey bees, P450s are involved in xenobiotic detoxification (Johnson et al., 2009; Berenbaum and Johnson, 2015). From a wider perspective, Chikungunya virus infection specifically activates the MAPK signaling pathways in humans (Varghese et al., 2016) and is a stress-responsive part of the intestinal innate immunity of *C. elegans* (Sakaguchi et al., 2004). Recent report showed that MAPK may play a role in regulating certain P450 genes (Yang et al., 2020). On the other hand, IAPV infection presumably causes major deregulation of the cellular homeostasis through the cooption of the cellular machinery to replicate itself (Boncristiani et al., 2013). Thus, deregulation of ribosome biosynthesis (Figure 3) and major energetic pathways is not surprising. Interestingly, transcriptomic analyses of Colony Collapse Disorder, which had been associated with IAPV (Cox-Foster et al., 2007), have shown similar immune responses (Johnson et al., 2009; Aufauvre et al., 2014). The methylomic profile of pupae 48 h after IAPV infection indicated genes involved in anti-parasitoid immune response (Howell et al., 2012; Haddad et al., 2016) to be affected. One of these genes that overlaps with a previous genomic study of honey bee immune genes (Evans et al., 2006) is Peroxin 23, a protein involved in peroxisomal protein import and associated with autophagosome formation in *Drosophila* muscles and central nervous system (Hazelett et al., 2012; Zirin et al., 2015). Compared to a previous report of the 5 mC methylomic profiles of honey bee adults infected with IAPV (Galbraith et al., 2015), our analysis revealed only one overlapping gene (GB51998), which is related to ATP binding, circadian regulation of gene expression systems (Claridge-Chang et al., 2001), and muscle morphogenesis and function in *Drosophila* (Schnorrer et al., 2010).

The list of identified DMRs linked to genes associated with ATP binding, phagosome, fatty acid degradation, RNA transport, and RNA degradation. Lipid metabolism, signaling, and biosynthesis are related to virus-host interactions (Chukkapalli et al., 2012). Fatty acid degradation and metabolism also responded to cold stress in other insects and might indicate increased energetic demands of stressed organisms (Vermeulen et al., 2013). Alternatively, IAPV may remodel the host cells by hijacking the lipid signaling pathways and synthesis process for its own replication, similar to virus manipulation of host RNA replication and transport mechanisms (Nagy and Pogany, 2012; Boncristiani et al., 2013).

Our study only represents a first step in the understanding of the intricate interplay of viruses and their honey bee host’s physiology. Future research needs to characterize the temporal transcriptome trends in more detail and tissue-specificity. Pupae are particularly relevant but also challenging because the ongoing metamorphosis entails drastic hormonal and presumably transcriptional changes even in the absence of disease. The discovered functional pathways need to be functionally investigated, particularly the putative 5 mC methylation control of immune genes.

## Materials and Methods

### Bee Samples and Virus Preparation

All honey bee samples were acquired from unselected experimental hives in the research apiary of the University of North Carolina Greensboro. A previously prepared and characterized IAPV solution in PBS was used for inoculations (Boncristiani et al., 2013). White-eyed pupae were either injected with PBS solution as control treatment or with 10^4^ genome copies of IAPV in 1.0 μl of the viral suspension per pupa. Pupae were maintained on folder filter papers in a sterile lab incubator at 32°C and 60% R.H. After 5, 20, and 48 h of injections, each individual pupal sample was flash-frozen in liquid nitrogen and then stored in a −80°C freezer. Figure 1 illustrates the experimental design.

### RNA-Seq Library Preparation and Sequencing

Treatment control (PBS-injection) and IAPV-infected pupae were compared across three time points (5, 20, and 48 h). Three pupae were collected for each treatment and each time point for a total of 18 samples. Dual extraction of nucleic acid (RNA and DNA) from each pupa was carried out using the MasterPure dual extraction kit (Epicenter, MC85200). Briefly, whole pupae were individually homogenized and lysed first by using a micropestle. The lysed cells were mixed with extraction buffer and centrifuged to remove proteins and undesired debris of the cell. Subsequent RNA and DNA fractions were treated with DNase and RNase accordingly. RNA-seq library preparation and sequencing were performed by the Genomic Sciences Laboratory of the North Carolina State University. Total RNA from each pupa was used to generate tagged Illumina libraries, using the NEBNext^®^ RNA Library Prep Kit (New England Biolab). All RNA-seq libraries were sequenced in two flow cells on the Illumina Next-Seq 500 (paired-end 150 bp length reads).

### BS-Seq Library Preparation and Sequencing

Bisulfite (BS) conversion of the genomic DNA from the above-mentioned dual extraction of each sample was carried out by using the EZ DNA Methylation-Lightning Kit (Zymo Research, D5030, Irvine, CA, United States). BS-converted DNA (100 ng) of each sample was used for preparing the BS-seq library via the TruSeq DNA Methylation Kit (Illumina, EGMK91324, San Diego, CA, United States). The quality of the libraries was checked using Qubit assays (Q32850, Life Technologies) and with the 2100 Bioanalyzer (Agilent Technologies, Inc.). The pooled 18 libraries were run on two replicate lanes of the Illumina NextSeq 500 (150 bp paired-end reads) by the Genomic Sciences Laboratory at NCSU.

### Bioinformatic Analyses

Overall quality control analysis of all resulting data was carried out by using FastQC.^1^ Raw data were trimmed by Trimmomatic (java -jar PE -phred33 ILLUMINACLIP:TruSeq3-PE.fa:2:30:10 MINLEN:36). TopHat2 v.2.0.12 (Kim et al., 2013) was used to align trimmed RNA-seq reads to the *Apis mellifera* 4.5 reference genome. General alignment parameters were chosen to allow three mismatches per segment, 12 mismatches per read, and gaps of up to 3 bp (tophat –read-mismatches 12 –segment-mismatches 3 –read-gap-length 3 –read-edit-dist 15 –b2-sensitive) to minimize the possibility that small sequence differences of our samples with the reference genome would bias expression estimates. These settings resulted in the successful alignment of 35% of raw reads (117–143 million per sample). Reconstructed transcripts were aligned with Cufflinks (v2.2.1) to the *Apis mellifera* OGS 3.2 allowing gaps of up to 1 bp (cufflinks –overlap-radius 1 –library-type fr-firststrand). To annotate promoters and transcription starts, we employed the “cuffcompare” command using OGS3.2 gff file (cuffcompare -s -CG -r).

Cuffdiff2 (v.2.1.1) (Kim et al., 2013) was used to test for differential expression between IAPV and control groups (*n* = 3 pupae treated as biological replicates at each of the 3 different time points) with the multiple mapping correction and a false discovery rate threshold set at 0.05. All three IAPV groups (IAPV_5h, IAPV_20h, and IAPV_48h) were compared to their corresponding sham-control groups (PBS_5h, PBS_20h, PBS_48h) to identify differentially expressed genes (DEGs). The data were explored and visualized with CummeRbundv.2.0.0^2^ and custom R scripts (Rsoftwarev.2.15.0).^3^ Differentially expressed genes with similar expression temporal dynamics were analyzed by cufflinks in R. Total of output of gene numbers is set to be 10 (for example, consider gene GB49890 (cyp6as5), we can find nine other genes in the database with similar expression patterns, then plot the expression patterns in Supplementary Figure 7).

Whole genome BS-Seq analysis was carried out by trimming the sequences using trim_galore (version 0.4.1)^4^ (trim_galore./file1_R1_001.fastq.gz./file1_R2_001.fastq.gz -q 20 –paired –phred33 -o). The Bismark tool (Krueger and Andrews, 2011) (bismark –bowtie2 –path_to_bowtie -p 8 path_to_genome_files/–1/file1 –2/file2) was employed for whole genome alignment. Brief procedures included Genome Conversion, Genome Alignment, Deduplication, and Methylation calls. Differentially methylated regions and genes (DMRs) were produced by methylKit in R (version 1.3.8) with percent methylation difference larger than 10% and *q* < 0.01 for the difference (Akalin et al., 2012).

The reference genome and official gene set were the same as described before. The sequencing raw data are published at the NCBI database (Sequence Read Archive (SRA) submission: SUB3404557, BioProject: PRJNA429508). All codes for the bioinformatic analyses are online.^5^

To determine overlap between DEG lists, either up- or down-regulated genes were compared between different time points. This directional overlap analysis avoids artifacts that can confound undirected DEG comparisons (Lawhorn et al., 2018). The analysis was compared focusing on DEGs with a more than 8-fold difference, and with DMRs (either hyper- or hypo-methylated). All overlap analyses were performed with hypergeometric tests to calculate the *p*-value for overlap based on the cumulative distribution function (CDF) of the hypergeometric distribution as published previously (Li-Byarlay et al., 2013). GO Mann-Whitney U tests were carried out on the gene expression data using codes published in Github.^6^ These analyses search for patterns of up- or down-regulation in genes associated with particular GO-terms based on their signed, uncorrected *p*-values regardless of significance thresholds (Wright et al., 2015).

For differentially transcript usage or spliced genes, transcript-level counts were quantified using Salmon1 v1.2.1 (Patro et al., 2017) against the A. mellifera HAv3.1 annotation. Viral RNAs were included in the transcriptome used for alignment but were omitted from isoform analysis. Salmon results were imported directly into the IsoformSwitchAnalyzeR R package (Vitting-Seerup and Sandelin, 2017; Vitting-Seerup and Sandelin, 2019), which normalizes transcript counts based on abundances and uses the TMM method to adjust effective library size. The version of IsoformSwitchAnalyzeR used was 1.11.3, obtained from GitHub commit 5a7ab4a due to a bug in version 1.11.2 on Bioconductor. Although CDS were annotated in the GFF file for A. mellifera, these caused problems in IsoformSwitchAnalyzeR due to inclusion of stop codons, as well as some transcripts having no annotated CDS or CDS that were obviously incorrect. Therefore, the analyzeORF function was used to predict open reading frames *de novo* from the transcript sequences, which in the vast majority of cases resulted in agreement with the published annotation. Nucleotide and amino acid sequences for all transcripts for significant genes were then exported for analysis with external tools: CPAT9 was used to determine whether a transcript was likely to be coding (Wang et al., 2013). It was run from the webserver using the *Drosophila* (dm3, BDGP release 5) assembly. A coding probability of 0.7 was used as the cutoff for classifying a transcript as coding or non-coding. Pfam10 (Punta et al., 2012) version 32.0 was used to predict protein domains, using pfam_scan v1.6 run on the Biocluster.

### Statistical Analysis of Differential Transcript Usage

A design matrix was constructed in which the condition was the combination of treatment and time point (six conditions total), and DWV infection (determined by alignment of reads to the DWV mRNA) was included as a covariate. Sample degradation was excluded as a covariate as it introduced singularity into the model at 48 h. The contrasts tested were IAPV_5h – PBS_5h, IAPV_20h – PBS_20h, and IAPV_48h – PBS_48h. The preFilter function in IsoformSwitchAnalyzeR was used with default settings to filter transcripts and genes. Genes were removed if they only had one isoform or if they did not have at least 1 RPKM in at least one condition. Transcripts were removed if they had no expression in any condition. After filtering, 15,808 isoforms belonging to 4,667 genes remained.

Differential transcript usage (DTU) was assessed using DEXSeq from within IsoformSwitchAnalyzeR (Anders et al., 2012; Ritchie et al., 2015; Vitting-Seerup and Sandelin, 2019). Genes were only retained for further analysis if they had an FDR < 0.05 for DTU, and if isoform usage (proportion of counts from a gene belonging to a given isoform) changed by at least 0.1 for at least two isoforms in opposite directions (i.e., the isoforms “switched”).

### Enrichment of Overlapping Genes Among Different Studies

To test whether our DEGs and DMRs had significant overlap with known immune genes, these lists were compared to published immune genes (Evans et al., 2006) using hypergeometric tests. Additionally, a directional overlap analysis was performed on our DEGs (separated by up- or down-regulation) with a previous RNA-seq study of IAPV infection in adult honey bee workers (Galbraith et al., 2015).

To identify potential motifs that are targeted for epigenetic modification after IAPV infection, differentially methylated regions (DMR) were analyzed post 5, 20, and 48 h IAPV infection generating 6-mer sequence logos with the online tool weblogo.^7^

## Data Availability Statement

The sequencing raw data are published at the NCBI database (Sequence Read Archive (SRA) submission: SUB3404557, BioProject: PRJNA429508).

## Author Contributions

HL-B, MS, DT, and OR conceptualized the scientific idea and aims. HB performed the viral infection experiments. HL-B prepared the samples and performed sequencing experiments and bioinformatics analyses. GH provided assistance on the computational techniques on High Performance Cluster. JH and LC provided additional analysis of GO and splicing. HL-B wrote the manuscript with all co-authors input. All authors contributed to the article and approved the submitted version.

## Conflict of Interest

The authors declare that the research was conducted in the absence of any commercial or financial relationships that could be construed as a potential conflict of interest.

## Footnotes

1 http://www.bioinformatics.babraham.ac.uk/projects/fastqc/

2 http://compbio.mit.edu/cummeRbund/

3 http://www.r-project.org/

4 http://www.bioinformatics.babraham.ac.uk/projects/trim_galore/

5 https://www.researchgate.net/publication/343237015_RNAseq_and_BSseq_codes_for_public

6 https://github.com/z0on/GO_MWU

7 https://weblogo.berkeley.edu

## References

[B1] Akalin A. Kormaksson M. Li S. Garrett-Bakelman F. E. Figueroa M. E. Melnick A. (2012). methylKit: a comprehensive R package for the analysis of genome-wide DNA methylation profiles. *Genome Biol.* 13:R87. 10.1186/gb-2012-13-10-r87 PMC3491415 23034086

[B2] Allis C. D. Jenuwein T. (2016). The molecular hallmarks of epigenetic control. *Nat. Rev. Genet.* 17 487–500. 10.1038/nrg.2016.59 27346641

[B3] Anders S. Reyes A. Huber W. (2012). Detecting differential usage of exons from RNA-seq data. *Genome Res.* 22 2008–2017. 10.1101/gr.133744.111 22722343 PMC3460195

[B4] Arvey A. Tempera I. Tsai K. Chen H.-S. Tikhmyanova N. Klichinsky M. (2012). An atlas of the epstein-barr virus transcriptome and epigenome reveals host-virus regulatory interactions. *Cell Host Microbe* 12 233–245. 10.1016/j.chom.2012.06.008 22901543 PMC3424516

[B5] Aufauvre J. Misme-Aucouturier B. Viguès B. Texier C. Delbac F. Blot N. (2014). Transcriptome analyses of the honeybee response to *Nosema ceranae* and insecticides. *PLoS One* 9:e91686. 10.1371/journal.pone.0091686 24646894 PMC3960157

[B6] Bailey L. Gibbs A. Woods R. (1963). Two viruses from adult honey bees (*Apis mellifera* Linnaeus). *Virology* 21 390–395. 10.1016/0042-6822(63)90200-9 14081363

[B7] Berenbaum M. R. Johnson R. M. (2015). Xenobiotic detoxification pathways in honey bees. *Curr. Opin. Insect Sci.* 10 51–58. 10.1016/j.cois.2015.03.005 29588014

[B8] Bewick A. J. Vogel K. J. Moore A. J. Schmitz R. J. (2017). Evolution of DNA methylation across insects. *Mol. Biol. Evol.* 34 654–665. 28025279 10.1093/molbev/msw264 PMC5400375

[B9] Boncristiani H. F. Evans J. D. Chen Y. Pettis J. Murphy C. Lopez D. L. (2013). In-vitro infection of pupae with Israeli Acute Paralysis Virus suggests variation for susceptibility and disturbance of transcriptional homeostasis in honey bees (*Apis mellifera*). *PLoS One* 8:e73429. 10.1371/journal.pone.0073429 24039938 PMC3764161

[B10] Bonning B. C. Miller W. A. (2010). Dicistroviruses. *Annu. Rev. Entomol.* 55 129–150. 10.1146/annurev-ento-112408-085457 19961327

[B11] Brutscher L. M. Daughenbaugh K. F. Flenniken M. L. (2015). Antiviral defense mechanisms in honey bees. *Curr. Opin. Insect Sci.* 10 71–82. 10.1016/j.cois.2015.04.016 26273564 PMC4530548

[B12] Brutscher L. M. Daughenbaugh K. F. Flenniken M. L. (2017). Virus and dsRNA-triggered transcriptional responses reveal key components of honey bee antiviral defense. *Sci. Rep.* 7:6448. 10.1038/s41598-017-06623-z PMC5526946 28743868

[B13] Burggren W. W. (2017). Epigenetics in insects: mechanisms, phenotypes and ecological and evolutionary implications. *Adv. Insect Physiol.* 53 1–30. 10.1016/bs.aiip.2017.04.001

[B14] Calderone N. W. (2012). Insect pollinated crops, insect pollinators and US agriculture: trend analysis of aggregate data for the period 1992–2009. *PLoS One* 7:e37235. 10.1371/journal.pone.0037235 22629374 PMC3358326

[B15] Chan C. Y. Low J. Z. H. Gan E. S. Ong E. Z. Zhang S. L.-X. Tan H. C. (2019). Antibody-dependent dengue virus entry modulates cell intrinsic responses for enhanced infection. *mSphere* 4:e00528-19. 10.1128/mSphere.00528-19 PMC6751492 31533998

[B16] Chen Y. P. Pettis J. S. Corona M. Chen W. P. Li C. J. Spivak M. (2014). Israeli acute paralysis virus: epidemiology, pathogenesis and implications for honey bee health. *PLoS Pathog.* 10:e1004261. 10.1371/journal.ppat.1004261 25079600 PMC4117608

[B17] Chen Y. P. Siede R. (2007). “Honey bee viruses,” in *Advances in Virus Research*, Vol. 70 eds Maramorosch K. Shatkin A. J. Murphy F. A. (Salt Lake City, UT: Academic Press), 33–80. 10.1016/S0065-3527(07)70002-7 17765703

[B18] Chukkapalli V. Heaton N. S. Randall G. (2012). Lipids at the interface of virus–host interactions. *Curr. Opin. Microbiol.* 15 512–518. 10.1016/j.mib.2012.05.013 22682978 PMC3424344

[B19] Cirimotich C. M. Dong Y. Garver L. S. Sim S. Dimopoulos G. (2010). Mosquito immune defenses against *Plasmodium* infection. *Dev. Comp. Immunol.* 34 387–395. 10.1016/j.dci.2009.12.005 20026176 PMC3462653

[B20] Claridge-Chang A. Wijnen H. Naef F. Boothroyd C. Rajewsky N. Young M. W. (2001). Circadian regulation of gene expression systems in the *Drosophila* head. *Neuron* 32 657–671. 10.1016/s0896-6273(01)00515-3 11719206

[B21] Cornman R. S. Boncristiani H. Dainat B. Chen Y. Weaver D. Evans J. D. (2013). Population-genomic variation within RNA viruses of the Western honey bee, *Apis mellifera*, inferred from deep sequencing. *BMC genomics.* 14:154. 10.1186/1471-2164-14-154 23497218 PMC3599929

[B22] Cornman R. S. Tarpy D. R. Chen Y. Jeffreys L. Lopez D. Pettis J. S. (2012). Pathogen webs in collapsing honey bee colonies. *PLoS One* 7:e43562. 10.1371/journal.pone.0043562 22927991 PMC3424165

[B23] Cox-Foster D. L. Conlan S. Holmes E. C. Palacios G. Evans J. D. Moran N. A. (2007). A metagenomic survey of microbes in honey bee colony collapse disorder. *Science* 318 283–287. 10.1126/science.1146498 17823314

[B24] Dantas Machado A. C. Zhou T. Rao S. Goel P. Rastogi C. Lazarovici A. (2015). Evolving insights on how cytosine methylation affects protein–DNA binding. *Brief. Funct. Genomics* 14 61–73. 10.1093/bfgp/elu040 25319759 PMC4303714

[B25] De Maio F. A. Risso G. Iglesias N. G. Shah P. Pozzi B. Gebhard L. G. (2016). The dengue virus NS5 protein intrudes in the cellular spliceosome and modulates splicing. *PLoS Pathog.* 12:e1005841. 10.1371/journal.ppat.1005841 27575636 PMC5004807

[B26] De Monerri N. C. S. Kim K. (2014). Pathogens hijack the epigenome: a new twist on host-pathogen interactions. *Am. J. Pathol.* 184 897–911. 24525150 10.1016/j.ajpath.2013.12.022 PMC3970002

[B27] Di Prisco G. Pennacchio F. Caprio E. Boncristiani H. F. Jr. Evans J. D. Chen Y. (2011). *Varroa destructor* is an effective vector of Israeli acute paralysis virus in the honeybee, *Apis mellifera*. *J. Gen. Virol.* 92 151–155. 10.1099/vir.0.023853-0 20926637

[B28] Dostert C. Jouanguy E. Irving P. Troxler L. Galiana-Arnoux D. Hetru C. (2005). The Jak-STAT signaling pathway is required but not sufficient for the antiviral response of drosophila. *Nat. Immunol.* 6 946–953. 10.1038/ni1237 16086017

[B29] Dubois J. Terrier O. Rosa-Calatrava M. (2014). Influenza viruses and mRNA splicing: doing more with less. *mBio* 5:e00070-14. 10.1128/mBio.00070-14 PMC4030477 24825008

[B30] Edwards T. M. Myers J. P. (2007). Environmental exposures and gene regulation in disease etiology. *Environ. Health Perspect.* 115 1264–1270. 10.1289/ehp.9951 17805414 PMC1964917

[B31] Evans J. D. Aronstein K. Chen Y. P. Hetru C. Imler J. L. Jiang H. (2006). Immune pathways and defence mechanisms in honey bees *Apis mellifera*. *Insect Mol. Biol.* 15 645–656. 10.1111/j.1365-2583.2006.00682.x 17069638 PMC1847501

[B32] Fang J. Hao Q. Liu L. Li Y. Wu J. Huo X. (2012). Epigenetic changes mediated by microRNA miR29 activate cyclooxygenase 2 and lambda-1 interferon production during viral infection. *J. Virol.* 86 1010–1020. 10.1128/jvi.06169-11 22072783 PMC3255816

[B33] Galbraith D. A. Yang X. Niño E. L. Yi S. Grozinger C. Schneider D. S. (2015). Parallel epigenomic and transcriptomic responses to viral infection in honey bees (*Apis mellifera*). *PLoS Pathog.* 11:e1004713. 10.1371/journal.ppat.1004713 25811620 PMC4374888

[B34] Gardy J. L. Loman N. J. (2018). APPLICATIONS OF NEXT-GENERATION SEQUENCING Towards a genomics-informed, real-time, global pathogen surveillance system. *Nat. Rev. Genet.* 19 9–20. 10.1038/nrg.2017.88 29129921 PMC7097748

[B35] Gisder S. Genersch E. (2017). Viruses of commercialized insect pollinators. *J. Invertebr. Pathol.* 147 51–59. 10.1016/j.jip.2016.07.010 27498219

[B36] Glastad K. M. Gokhale K. Liebig J. Goodisman M. A. (2016). The caste-and sex-specific DNA methylome of the termite *Zootermopsis nevadensis*. *Sci. Rep.* 6:37110. 10.1038/srep37110 PMC5111047 27848993

[B37] Glastad K. M. Goodisman M. A. Yi S. V. Hunt B. G. (2015). Effects of DNA methylation and chromatin state on rates of molecular evolution in insects. *G3* 6 357–363. 10.1534/g3.115.023499 26637432 PMC4751555

[B38] Glastad K. M. Hunt B. G. Goodisman M. A. (2019). Epigenetics in insects: genome regulation and the generation of phenotypic diversity. *Annu. Rev. Entomol.* 64 185–203. 10.1146/annurev-ento-011118-111914 30285490

[B39] Glastad K. M. Hunt B. G. Goodisman M. A. D. (2014). Evolutionary insights into DNA methylation in insects. *Curr. Opin. Insect Sci.* 1 25–30. 10.1016/j.cois.2014.04.001 32846726

[B40] Goulson D. Nicholls E. Botias C. Rotheray E. L. (2015). Bee declines driven by combined stress from parasites, pesticides, and lack of flowers. *Science* 347:1255957. 10.1126/science.1255957 25721506

[B41] Grozinger C. M. Flenniken M. L. (2019). Bee viruses: ecology, pathogenicity, and impacts. *Annu. Rev. Entomol.* 64 205–226. 10.1146/annurev-ento-011118-111942 30629896

[B42] Haddad N. Mahmud Batainh A. Suleiman Migdadi O. Saini D. Krishnamurthy V. Parameswaran S. (2016). Next generation sequencing of *Apis mellifera syriaca* identifies genes for *Varroa* resistance and beneficial bee keeping traits. *Insect Sci.* 23 579–590. 10.1111/1744-7917.12205 25615619

[B43] Hazelett D. J. Chang J. C. Lakeland D. L. Morton D. B. (2012). Comparison of parallel high-throughput RNA sequencing between knockout of TDP-43 and its overexpression reveals primarily nonreciprocal and nonoverlapping gene expression changes in the central nervous system of Drosophila. *G3* 2 789–802. 10.1534/g3.112.002998 22870402 PMC3385985

[B44] Herb B. R. Wolschin F. Hansen K. D. Aryee M. J. Langmead B. Irizarry R. (2012). Reversible switching between epigenetic states in honeybee behavioral subcastes. *Nat. Neurosci.* 15 1371–1373. 10.1038/nn.3218 22983211 PMC3518384

[B45] Howell L. Sampson C. J. Xavier M. J. Bolukbasi E. Heck M. M. Williams M. J. (2012). A directed miniscreen for genes involved in the *Drosophila* anti-parasitoid immune response. *Immunogenetics* 64 155–161. 10.1007/s00251-011-0571-3 21947570

[B46] Huang H. Wu P. Zhang S. Shang Q. Yin H. Hou Q. (2019). DNA methylomes and transcriptomes analysis reveal implication of host DNA methylation machinery in BmNPV proliferation in *Bombyx mori*. *BMC genomics.* 20:736. 10.1186/s12864-019-6146-7 31615392 PMC6792228

[B47] Hunt B. G. Brisson J. A. Yi S. V. Goodisman M. A. D. (2010). Functional conservation of DNA methylation in the pea aphid and the honeybee. *Genome Biol. Evol.* 2 719–728. 10.1093/gbe/evq057 20855427 PMC2962555

[B48] Hunt B. G. Glastad K. M. Yi S. V. Goodisman M. A. D. (2013). The function of intragenic DNA methylation: insights from insect epigenomes. *Integr. Comp. Biol.* 53 319–328. 10.1093/icb/ict003 23509238

[B49] Johnson R. M. Evans J. D. Robinson G. E. Berenbaum M. R. (2009). Changes in transcript abundance relating to colony collapse disorder in honey bees (*Apis mellifera*). *Proc. Natl. Acad. Sci. U. S. A.* 106 14790–14795. 10.1073/pnas.0906970106 19706391 PMC2736458

[B50] Kim D. Pertea G. Trapnell C. Pimentel H. Kelley R. Salzberg S. L. (2013). TopHat2: accurate alignment of transcriptomes in the presence of insertions, deletions and gene fusions. *Genome Biol.* 14:R36. 10.1186/gb-2013-14-4-r36 PMC4053844 23618408

[B51] Krueger F. Andrews S. R. (2011). Bismark: a flexible aligner and methylation caller for Bisulfite-Seq applications. *Bioinformatics* 27 1571–1572. 10.1093/bioinformatics/btr167 21493656 PMC3102221

[B52] Kucharski R. Maleszka J. Foret S. Maleszka R. (2008). Nutritional control of reproductive status in honeybees via DNA methylation. *Science* 319 1827–1830. 10.1126/science.1153069 18339900

[B53] Kulhanek K. Steinhauer N. Rennich K. Caron D. M. Sagili R. R. Pettis J. S. (2017). A national survey of managed honey bee 2015–2016 annual colony losses in the USA. *J. Apic. Res.* 56 328–340. 10.1080/00218839.2017.1344496

[B54] Kusano M. Toyota M. Suzuki H. Akino K. Aoki F. Fujita M. (2006). Genetic, epigenetic, and clinicopathologic features of gastric carcinomas with the CpG island methylator phenotype and an association with Epstein–Barr virus. *Cancer* 106 1467–1479. 10.1002/cncr.21789 16518809

[B55] Kuss-Duerkop S. K. Westrich J. A. Pyeon D. (2018). DNA tumor virus regulation of host DNA methylation and its implications for immune evasion and oncogenesis. *Viruses* 10:82. 10.3390/v10020082 29438328 PMC5850389

[B56] Kuster R. D. Boncristiani H. F. Rueppell O. (2014). Immunogene and viral transcript dynamics during parasitic *Varroa destructor* mite infection of developing honey bee (*Apis mellifera*) pupae. *J. Exp. Biol.* 217 1710–1718. 10.1242/jeb.097766 24829325

[B57] Lawhorn C. M. Schomaker R. Rowell J. T. Rueppell O. (2018). Simple comparative analyses of differentially expressed gene lists may overestimate gene overlap. *J. Comput. Biol.* 25 606–612. 10.1089/cmb.2017.0262 29658777 PMC5998827

[B58] Lemaitre B. Hoffmann J. (2007). The host defense of *Drosophila melanogaster*. *Annu. Rev. Immunol.* 25 697–743. 17201680 10.1146/annurev.immunol.25.022106.141615

[B59] Li H. M. Buczkowski G. Mittapalli O. Xie J. Wu J. Westerman R. (2008). Transcriptomic profiles of *Drosophila melanogaster* third instar larval midgut and responses to oxidative stress. *Insect Mol. Biol.* 17 325–339. 10.1111/j.1365-2583.2008.00808.x 18651915

[B60] Li-Byarlay H. (2016). The function of DNA methylation marks in social insects. *Front. Ecol. Evol.* 4:57 10.3389/fevo.2016.00057

[B61] Li-Byarlay H. Li Y. Stroud H. Feng S. Newman T. C. Hou K. K. (2013). RNA interference knockdown of DNA methyl-transferase 3 affects gene alternative splicing in the honey bee. *Proc. Natl. Acad. Sci. U.S.A.* 110 12750–12755. 10.1073/pnas.1310735110 23852726 PMC3732956

[B62] Lyko F. Foret S. Kucharski R. Wolf S. Falckenhayn C. Maleszka R. (2010). The honey bee epigenomes: differential methylation of brain DNA in queens and workers. *PLoS Biol.* 8:e1000506. 10.1371/journal.pbio.1000506 21072239 PMC2970541

[B63] Maori E. Lavi S. Mozes-Koch R. Gantman Y. Peretz Y. Edelbaum O. (2007). Isolation and characterization of Israeli acute paralysis virus, a dicistrovirus affecting honeybees in Israel: evidence for diversity due to intra- and inter-species recombination. *J. Gen. Virol.* 88(Pt 12), 3428–3438. 10.1099/vir.0.83284-0 18024913

[B64] Maori E. Paldi N. Shafir S. Kalev H. Tsur E. Glick E. (2009). IAPV, a bee-affecting virus associated with Colony Collapse Disorder can be silenced by dsRNA ingestion. *Insect Mol. Biol.* 18 55–60. 10.1111/j.1365-2583.2009.00847.x 19196347

[B65] Marina R. J. Sturgill D. Bailly M. A. Thenoz M. Varma G. Prigge M. F. (2016). TET-catalyzed oxidation of intragenic 5-methylcytosine regulates CTCF-dependent alternative splicing. *EMBO J.* 35 335–355. 10.15252/embj.201593235 26711177 PMC4741300

[B66] Matilainen O. Quirós P. M. Auwerx J. (2017). Mitochondria and epigenetics–crosstalk in homeostasis and stress. *Trends Cell Biol.* 27 453–463. 10.1016/j.tcb.2017.02.004 28274652

[B67] McMenamin A. J. Genersch E. (2015). Honey bee colony losses and associated viruses. *Curr. Opin. Insect Sci.* 8 121–129. 10.1016/j.cois.2015.01.015 32846659

[B68] Merkling S. H. Bronkhorst A. W. Kramer J. M. Overheul G. J. Schenck A. Van Rij R. P. (2015). The epigenetic regulator G9a mediates tolerance to RNA virus infection in Drosophila. *PLoS Pathog.* 11:e1004692. 10.1371/journal.ppat.1004692 25880195 PMC4399909

[B69] Mukherjee K. Twyman R. M. Vilcinskas A. (2015). Insects as models to study the epigenetic basis of disease. *Prog. Biophys. Mol. Biol.* 118 69–78. 10.1016/j.pbiomolbio.2015.02.009 25778758

[B70] Munoz-Wolf N. Lavelle E. C. (2017). Hippo interferes with antiviral defences. *Nat. Cell Biol.* 19 267–269. 10.1038/ncb3502 28361938

[B71] Nagy P. D. Pogany J. (2012). The dependence of viral RNA replication on co-opted host factors. *Nat. Rev. Microbiol.* 10 137–149. 10.1038/nrmicro2692 22183253 PMC7097227

[B72] Opachaloemphan C. Yan H. Leibholz A. Desplan C. Reinberg D. (2018). Recent advances in behavioral (epi) genetics in eusocial insects. *Annu. Rev. Genet.* 52 489–510. 10.1146/annurev-genet-120116-024456 30208294 PMC6445553

[B73] Paschos K. Allday M. J. (2010). Epigenetic reprogramming of host genes in viral and microbial pathogenesis. *Trends Microbiol.* 18 439–447. 10.1016/j.tim.2010.07.003 20724161 PMC3089700

[B74] Patro R. Duggal G. Love M. I. Irizarry R. A. Kingsford C. (2017). Salmon provides fast and bias-aware quantification of transcript expression. *Nat. Methods* 14 417–419. 10.1038/nmeth.4197 28263959 PMC5600148

[B75] Pavet V. Quintero C. Cecchini N. M. Rosa A. L. Alvarez M. E. (2006). Arabidopsis displays centromeric DNA hypomethylation and cytological alterations of heterochromatin upon attack by *Pseudomonas syringae*. *Mol. Plant Microbe Interact.* 19 577–587. 10.1094/mpmi-19-0577 16776291

[B76] Punta M. Coggill P. C. Eberhardt R. Y. Mistry J. Tate J. Boursnell C. (2012). The Pfam protein families database. *Nucleic Acids Res.* 40 D290–D301. 22127870 10.1093/nar/gkr1065 PMC3245129

[B77] Ritchie M. E. Phipson B. Wu D. Hu Y. Law C. W. Shi W. (2015). limma powers differential expression analyses for RNA-sequencing and microarray studies. *Nucleic Acids Res.* 43:e47. 10.1093/nar/gkv007 25605792 PMC4402510

[B78] Runckel C. Flenniken M. L. Engel J. C. Ruby J. G. Ganem D. Andino R. (2011). Temporal analysis of the honey bee microbiome reveals four novel viruses and seasonal prevalence of known viruses, *Nosema*, and *Crithidia*. *PLoS One* 6:e20656. 10.1371/journal.pone.0020656 21687739 PMC3110205

[B79] Sakaguchi A. Matsumoto K. Hisamoto N. (2004). Roles of MAP kinase cascades in *Caenorhabditis elegans*. *J. Biochem.* 136 7–11. 10.1093/jb/mvh097 15269234

[B80] Saripalli G. Sharma C. Gautam T. Singh K. Jain N. Prasad P. (2020). Complex relationship between DNA methylation and gene expression due to Lr28 in wheat-leaf rust pathosystem. *Mol. Biol. Rep.* 47 1339–1360. 10.1007/s11033-019-05236-1 31873872

[B81] Schnorrer F. Schonbauer C. Langer C. C. H. Dietzl G. Novatchkova M. Schernhuber K. (2010). Systematic genetic analysis of muscle morphogenesis and function in *Drosophila*. *Nature* 464 287–291. 10.1038/nature08799 20220848

[B82] Shukla S. Kavak E. Gregory M. Imashimizu M. Shutinoski B. Kashlev M. (2011). CTCF-promoted RNA polymerase II pausing links DNA methylation to splicing. *Nature* 479 74–79. 10.1038/nature10442 21964334 PMC7398428

[B83] Tanca A. Deligios M. Addis M. F. Uzzau S. (2013). High throughput genomic and proteomic technologies in the fight against infectious diseases. *J. Infect. Dev. Ctries.* 7 182–190. 10.3855/jidc.3027 23492995

[B84] Tantillo G. Bottaro M. Di Pinto A. Martella V. Di Pinto P. Terio V. (2015). Virus infections of honeybees *Apis mellifera*. *Ital. J. Food Saf.* 4 157–168. 10.4081/ijfs.2015.5364 PMC5076640 27800411

[B85] Tian F. Zhan F. VanderKraats N. D. Hiken J. F. Edwards J. R. Zhang H. (2013). DNMT gene expression and methylome in Marek’s disease resistant and susceptible chickens prior to and following infection by MDV. *Epigenetics* 8 431–444. 10.4161/epi.24361 23538681 PMC3674052

[B86] Toyota M. Suzuki H. Yamashita T. Hirata K. Imai K. Tokino T. (2009). Cancer epigenomics: implications of DNA methylation in personalized cancer therapy. *Cancer Sci.* 100 787–791. 10.1111/j.1349-7006.2009.01095.x 19236379 PMC11159488

[B87] Toyota M. Yamamoto E. (2011). DNA methylation changes in cancer. *Prog. Mol. Biol. Transl. Sci.* 101 447–457. 21507361 10.1016/B978-0-12-387685-0.00014-7

[B88] Turner M. Diaz-Munoz M. D. (2018). RNA-binding proteins control gene expression and cell fate in the immune system. *Nat. Immunol.* 19 120–129. 10.1038/s41590-017-0028-4 29348497

[B89] Varghese F. S. Thaa B. Amrun S. N. Simarmata D. Rausalu K. Nyman T. A. (2016). The antiviral alkaloid berberine reduces chikungunya virus-induced mitogen-activated protein kinase signaling. *J. Virol.* 90 9743–9757. 10.1128/jvi.01382-16 27535052 PMC5068526

[B90] Vermeulen C. J. Sorensen P. Gagalova K. K. Loeschcke V. (2013). Transcriptomic analysis of inbreeding depression in cold-sensitive *Drosophila melanogaster* shows upregulation of the immune response. *J. Evol. Biol.* 26 1890–1902. 10.1111/jeb.12183 23944235

[B91] Vilcinskas A. (2017). The impact of parasites on host insect epigenetics. *Adv. Insect Physiol.* 53 145–165. 10.1016/bs.aiip.2017.05.001

[B92] Vitting-Seerup K. Sandelin A. (2017). The landscape of isoform switches in human cancers. *Mol. Cancer Res.* 15 1206–1220. 10.1158/1541-7786.mcr-16-0459 28584021

[B93] Vitting-Seerup K. Sandelin A. (2019). IsoformSwitchAnalyzeR: analysis of changes in genome-wide patterns of alternative splicing and its functional consequences. *Bioinformatics* 35 4469–4471. 10.1093/bioinformatics/btz247 30989184

[B94] Wang L. Park H. J. Dasari S. Wang S. Kocher J.-P. Li W. (2013). CPAT: coding-potential assessment tool using an alignment-free logistic regression model. *Nucleic Acids Res.* 41:e74. 10.1093/nar/gkt006 23335781 PMC3616698

[B95] Wang Y. Jorda M. Jones P. L. Maleszka R. Ling X. Robertson H. M. (2006). Functional CpG methylation system in a social insect. *Science* 314 645–647. 10.1126/science.1135213 17068262

[B96] Wang Y. Li-Byarlay H. (2015). Physiological and molecular mechanisms of nutrition in honey bees. *Adv. Insect Physiol.* 49 25–58. 10.1016/bs.aiip.2015.06.002

[B97] Wilson-Rich N. Spivak M. Fefferman N. H. Starks P. T. (2009). Genetic, individual, and group facilitation of disease resistance in insect societies. *Annu. Rev. Entomol.* 54 405–423. 10.1146/annurev.ento.53.103106.093301 18793100

[B98] Wright R. M. Aglyamova G. V. Meyer E. Matz M. V. (2015). Gene expression associated with white syndromes in a reef building coral, *Acropora hyacinthus*. *BMC Genomics* 16:371. 10.1186/s12864-015-1540-2 25956907 PMC4425862

[B99] Xu J. Grant G. Sabin L. R. Gordesky-Gold B. Yasunaga A. Tudor M. (2012). Transcriptional pausing controls a rapid antiviral innate immune response in *Drosophila*. *Cell Host Microbe* 12 531–543. 10.1016/j.chom.2012.08.011 23084920 PMC3479682

[B100] Yan H. Simola D. F. Bonasio R. Liebig J. Berger S. L. Reinberg D. (2014). Eusocial insects as emerging models for behavioural epigenetics. *Nat. Rev. Genet.* 15 677–688. 10.1038/nrg3787 25200663

[B101] Yang X. Deng S. Wei X. Yang J. Zhao Q. Yin C. (2020). MAPK-directed activation of the whitefly transcription factor CREB leads to P450-mediated imidacloprid resistance. *Proc. Natl. Acad. Sci. U.S.A.* 117 10246–10253. 10.1073/pnas.1913603117 32327610 PMC7229646

[B102] Zemach A. McDaniel I. E. Silva P. Zilberman D. (2010). Genome-wide evolutionary analysis of eukaryotic DNA methylation. *Science* 328 916–919. 10.1126/science.1186366 20395474

[B103] Zirin J. Nieuwenhuis J. Samsonova A. Tao R. Perrimon N. (2015). Regulators of autophagosome formation in *Drosophila* muscles. *PLoS Genet.* 11:e1005006. 10.1371/journal.pgen.1005006 25692684 PMC4334200

