# Supplementary figures and images for: Transcriptomic and Epigenomic Dynamics of Honey Bees in Response to Lethal Viral Infection
Source: Front Genet. 2020 Sep 24;11:566320. doi: 10.3389/fgene.2020.566320 (PMC7546774; doi:10.3389/fgene.2020.566320)

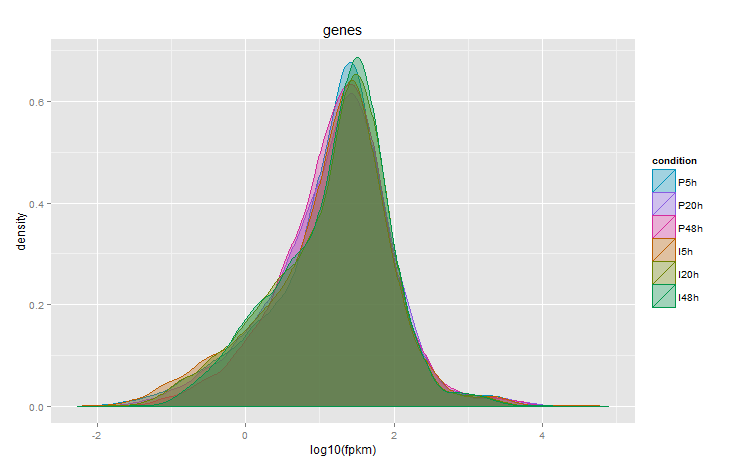

Supplement: Supplementary file 1 [file Image_1.PNG]

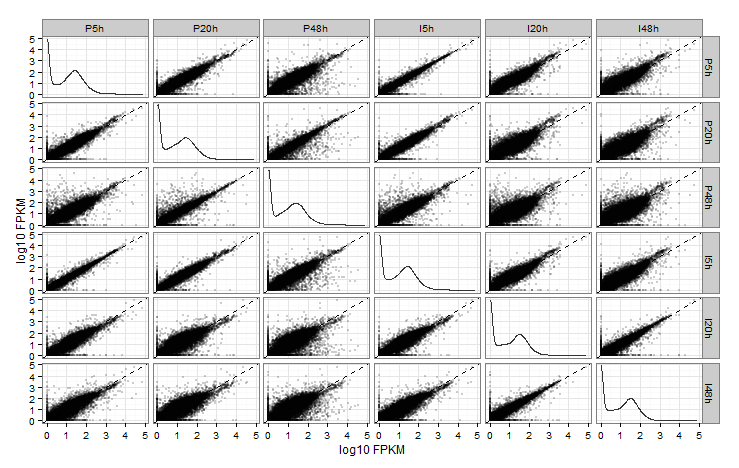

Supplement: Supplementary file 2 [file Image_2.PNG]

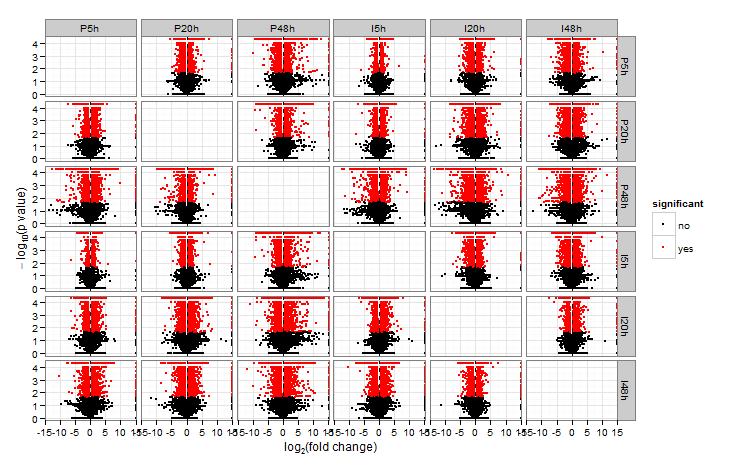

Supplement: Supplementary file 3 [file Image_3.PNG]

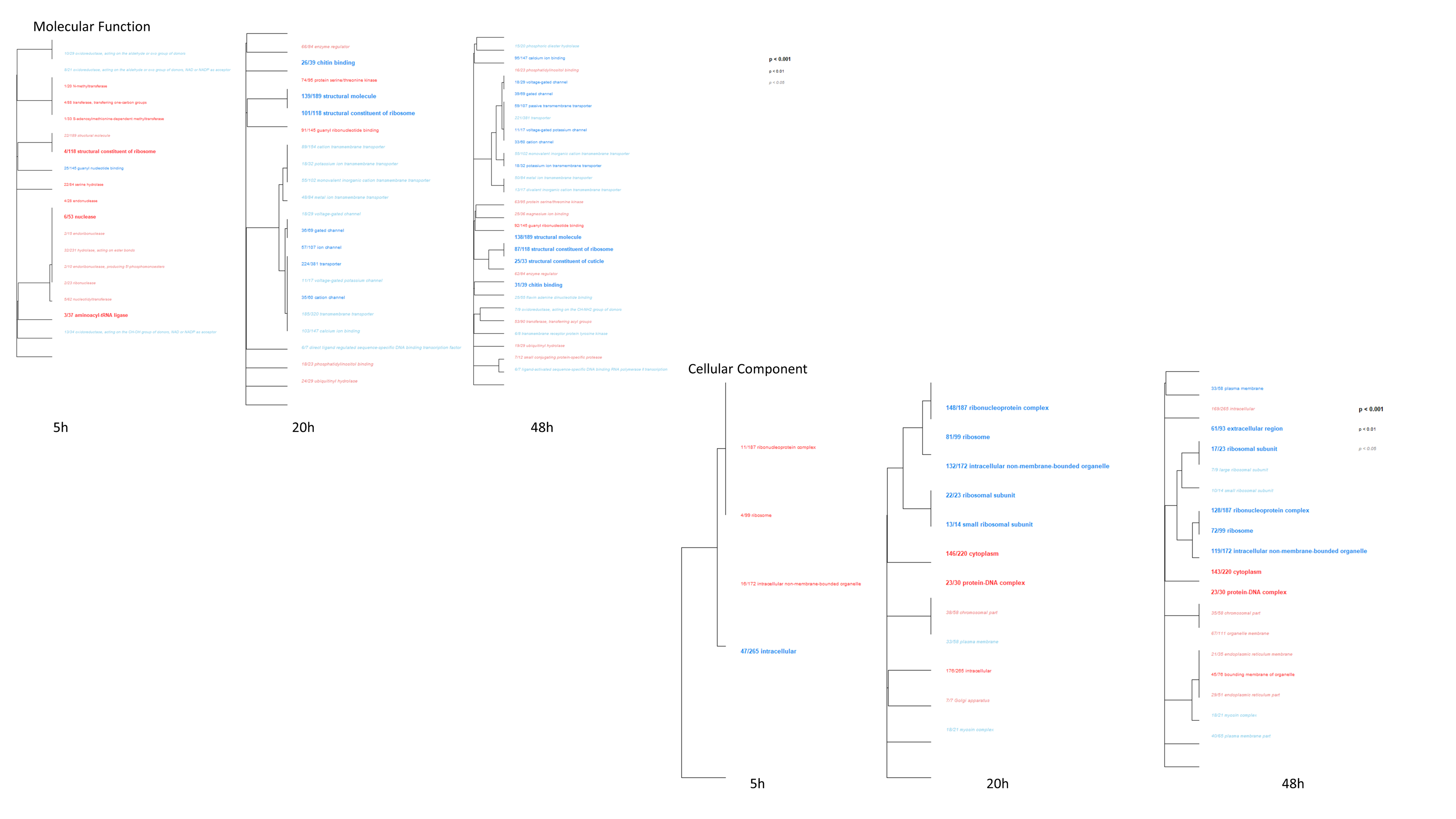

Supplement: Supplementary file 4 [file Image_4.tif]

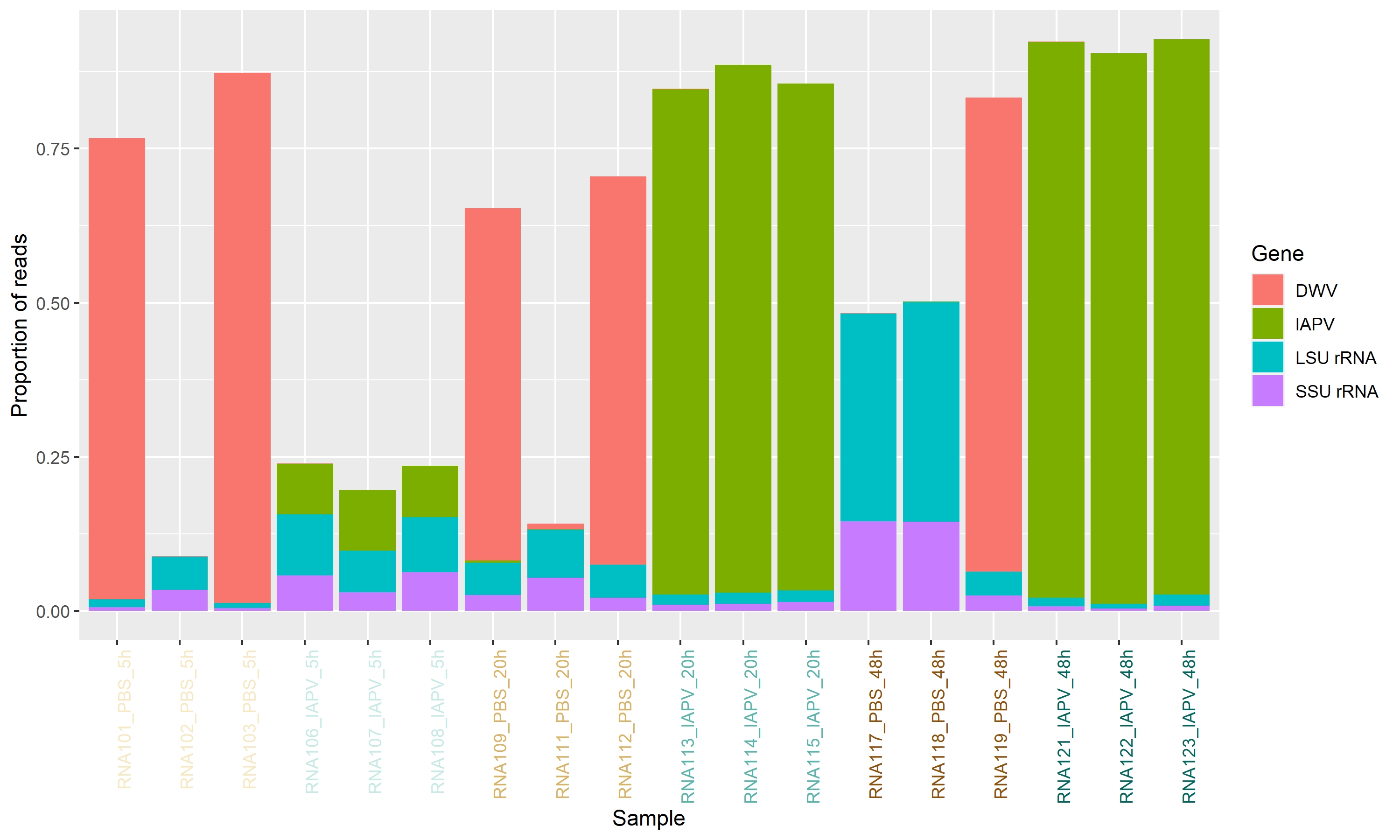

Supplement: Supplementary file 5 [file Image_5.jpeg]

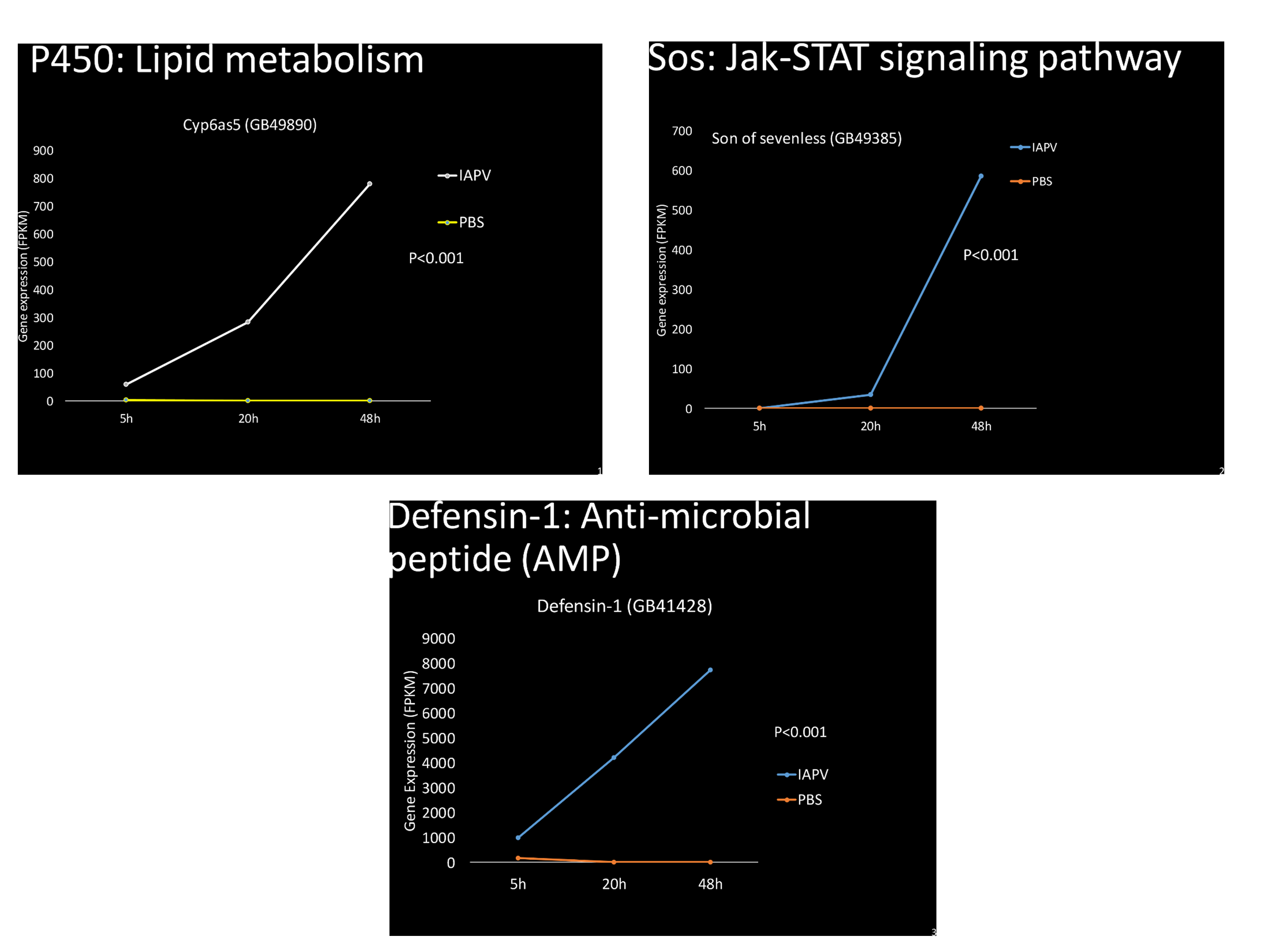

Supplement: Supplementary file 6 [file Image_6.tif]

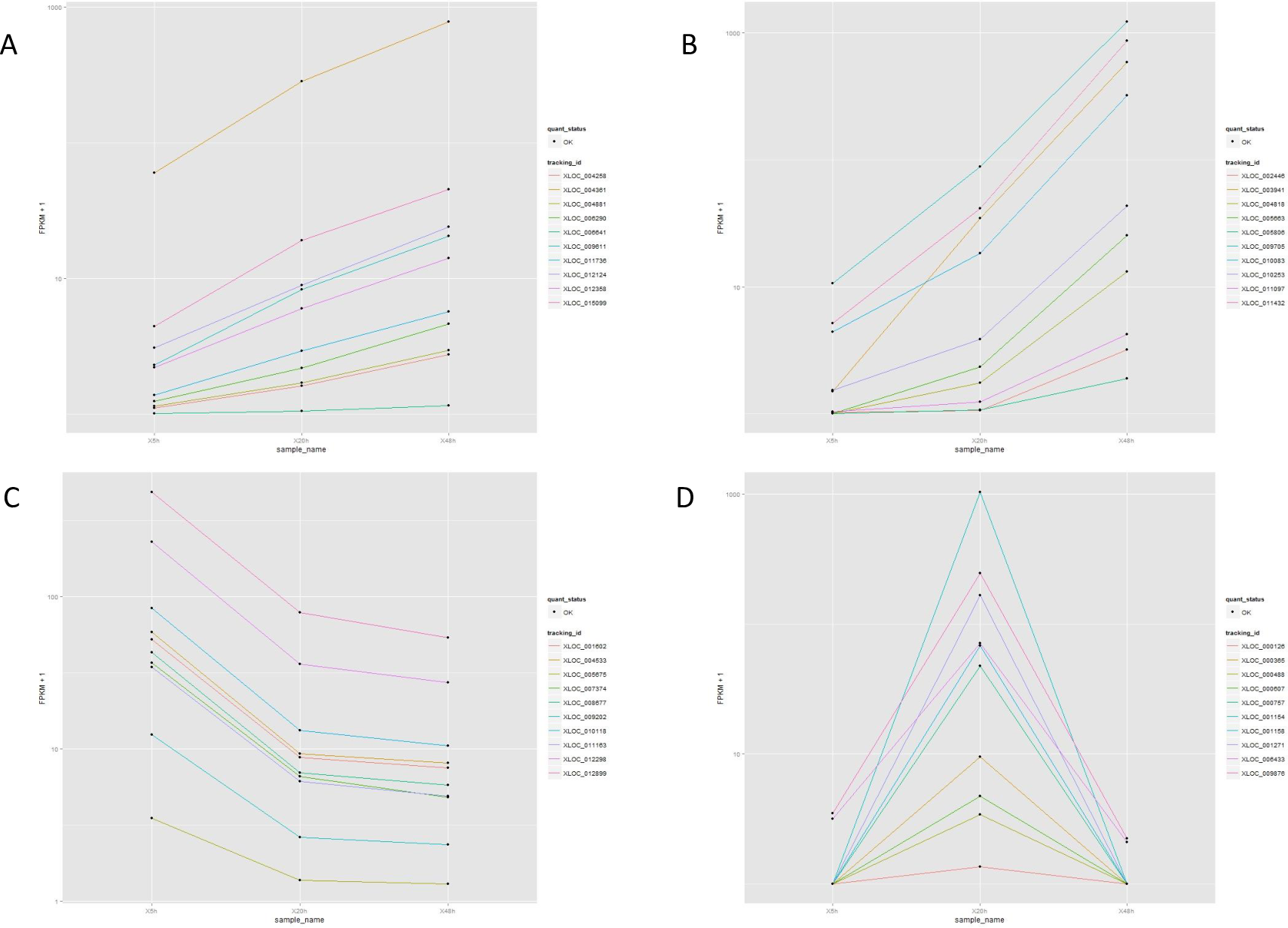

Supplement: Supplementary file 7 [file Image_7.tif]

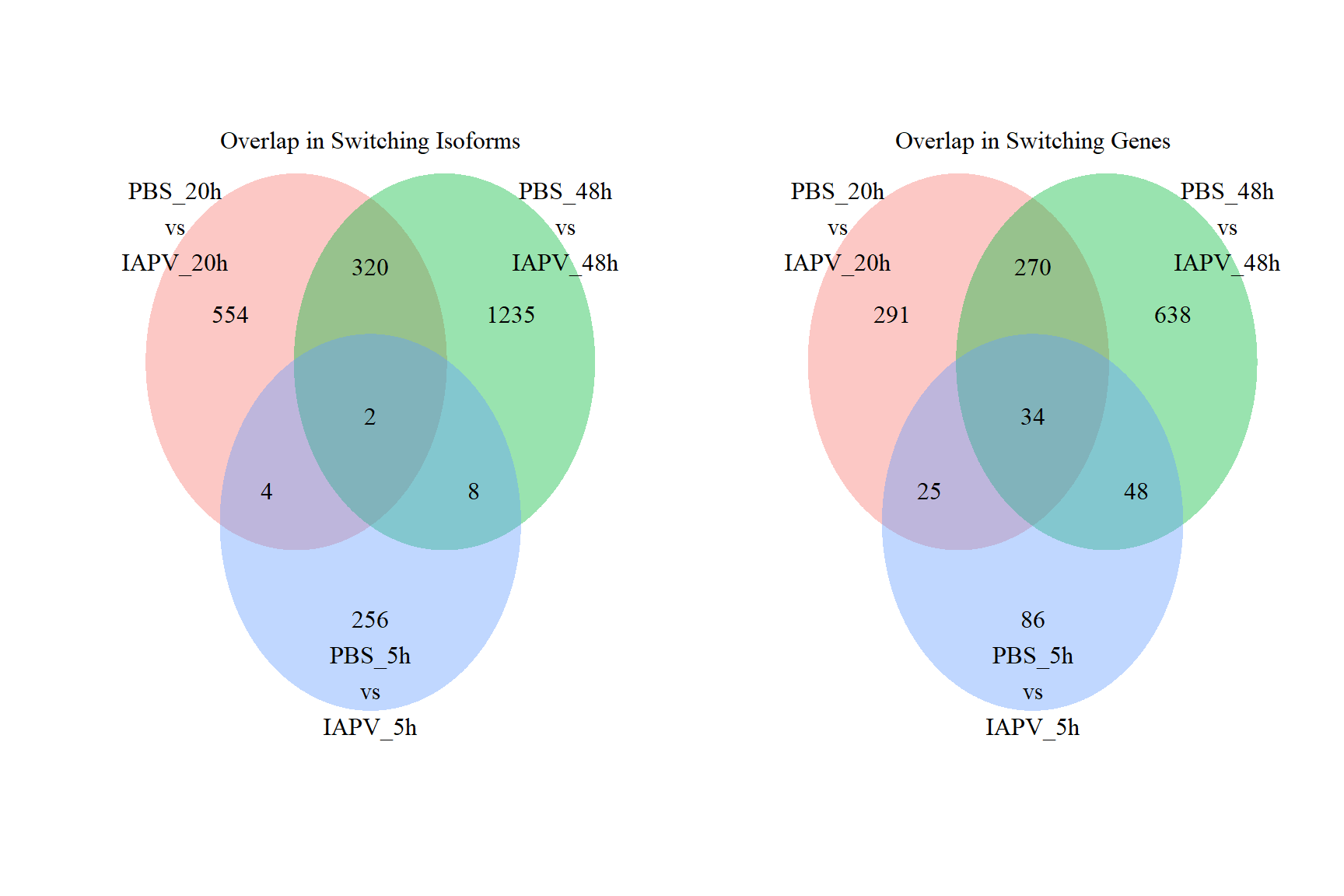

Supplement: Supplementary file 8 [file Image_8.png]

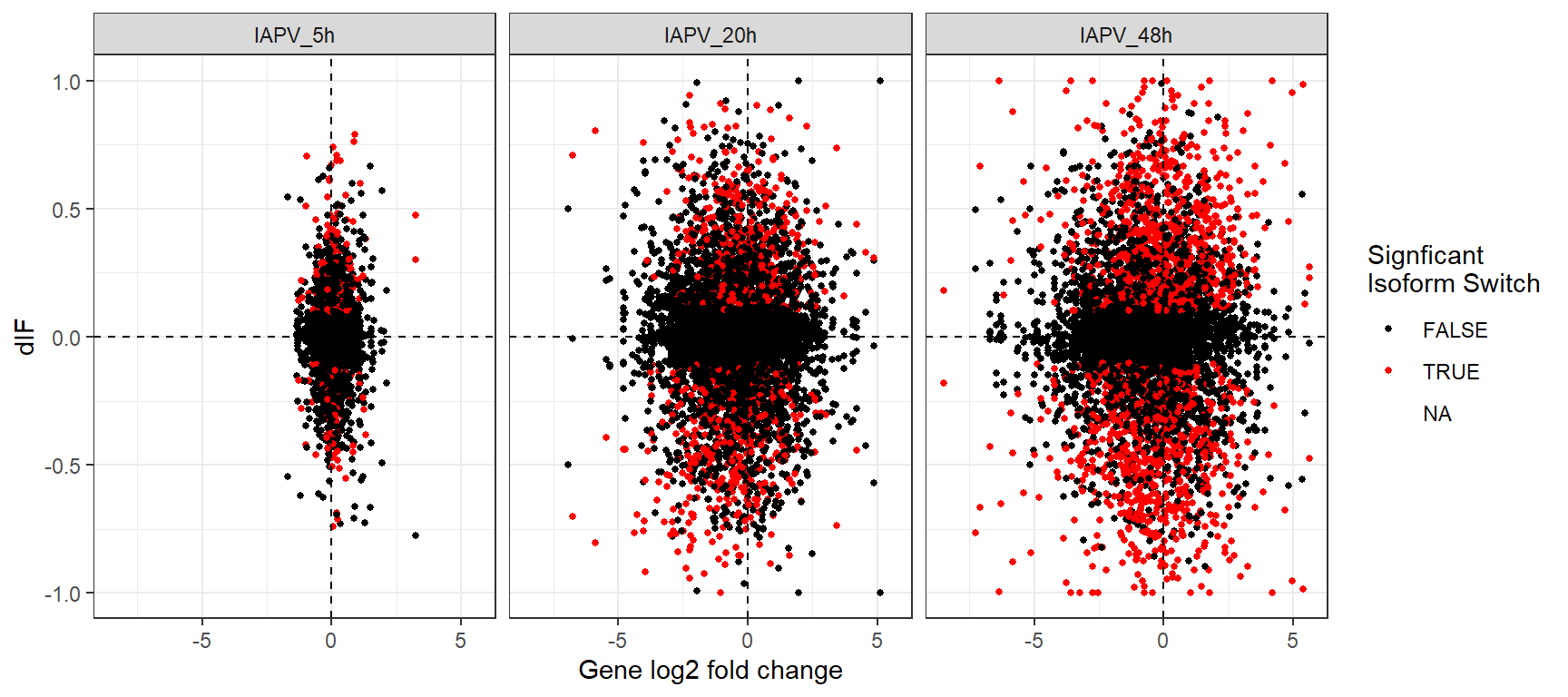

Supplement: Supplementary file 9 [file Image_9.png]

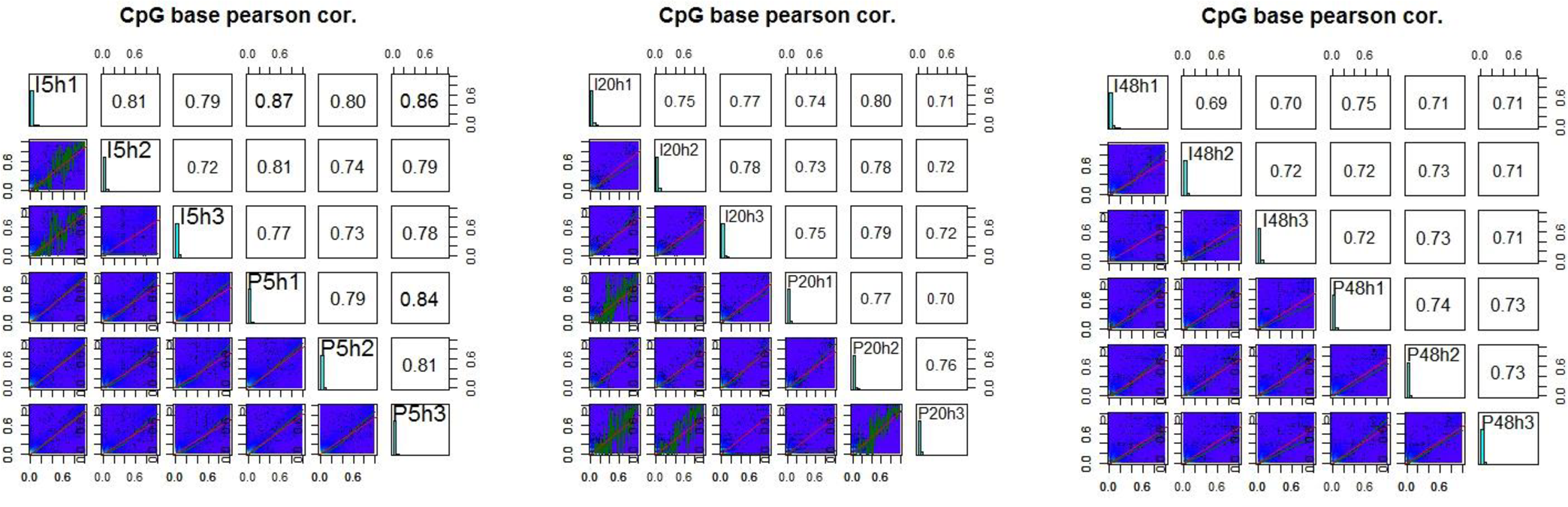

Supplement: Supplementary file 10 [file Image_10.tif]

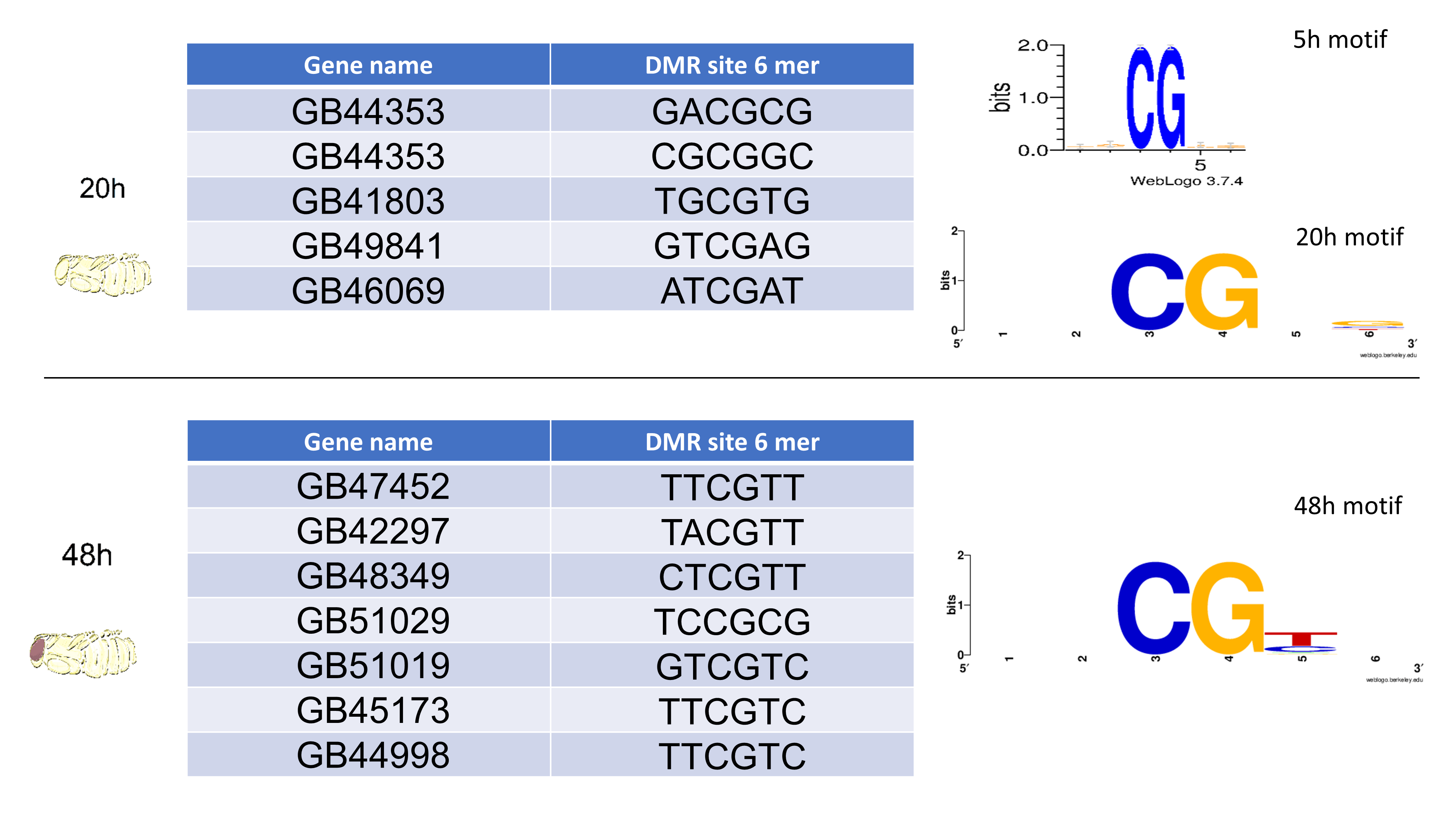

Supplement: Supplementary file 11 [file Image_11.tif]
