# Supplementary material for: Transcriptomic and Epigenomic Dynamics of Honey Bees in Response to Lethal Viral Infection
Source: Front Genet. 2020 Sep 24;11:566320. doi: 10.3389/fgene.2020.566320 (PMC7546774; doi:10.3389/fgene.2020.566320)
Supplement: Supplementary file 20 [file Data_Sheet_1.docx]

# Transcriptomic and epigenomic dynamics of honey bees in response to lethal viral infection

Li-Byarlay H, Boncristiani H, Howell G, Herman J, Clark L, Strand MK, Tarpy D, and Rueppell O

Supple Figure 1_Rplot: Rplot to show IAPV infections caused changes in gene expression after 5h, 20h, and 48h. The density plot showing all read counts of genes for each sample. P stands for PBS sham control samples. I stands for IAPV infected samples.

Supple Figure 2_scatterplot matrix: IAPV infections caused changes in gene expression after 5h, 20h, and 48h. A. The scatterplot matrix showing all gene expression of FPKM values (n=15,313 genes). P stands for PBS sham control samples. I stands for IAPV infected samples.

Supple Figure 3_volcano matrix: IAPV infections caused changes in gene expression after 5h, 20h, and 48h. The volcano matrix plot shows the genes differentially expressed (red) and not significantly expressed (black). P stands for PBS sham control samples. I stands for IAPV infected samples.

Supple Figure 4 GOMWU figures: GO Mann-Whitney U tests of DEGs to reveal their molecular functions and cellular components between IAPV and control samples after 5h, 20h, and 48h infection.

Supple Figure 5 virus and rRNA: A large number of IAPV reads was observed in the IAPV-inoculated pupae, with the proportion being similarly high at 20h and 48h. Additionally, five of the control samples had a large proportion of reads mapping to DWV, suggesting unintentional infection with this virus. Two covariates were added to the statistical model for detecting differential gene expression.

Supple Figure 6 DEG genes examples in one: Different gene expression patterns in *Cyp6as5*, *Son of Sevenless* (*SoS*), and Defensin-1, which displayed dramatic increases from 5h to 20h, and 20h to 48h infection.

Supple Figure 7 temporal genes. Gene expression profiles with similar temporal dynamic changes from our transcriptomic profiles. (A) Expression profiles of gene *Cyp6as5* (involved in lipid metabolism) and nine other genes up-regulated post 20h and 48h IAPV infection; (B) Expression profiles of gene *Son of Sevenless* (*SoS)* and nine other genes up-regulated post 20h and 48h IAPV infection; (C) Expression profiles of gene *epidermal growth factor receptor* (*EGFR)* and nine other genes down-regulated post IAPV infection; and (D) Expression profiles of gene *GB55029* and nine other genes up-regulated post IAPV infection 5h to 20h, then down-regulated post 20h-48h IAPV infection. X5h, X20h, X48h indicates samples from the treatment post 5h, 20h, and 48h IAPV infections.

Supple Figure 8 splicing genes overlap 5 20 48: Differential transcript usage (DTU) (FDR < 0.05) and if isoform usage (proportion of counts from a gene belonging to a given isoform) changed by at least 0.1 for at least two isoforms in opposite directions (i.e. the isoforms “switched”). Significant hits are summarized.

Supple Figure 9 sig splicing genes: The plot to show the gene fold change of significant isoform switch or alternative splicing after 5h, 20h, and 48h viral infections.

Supple Figure 10 methylkit correlation: The whole genome-wide Pearson correlation matrix for CpG base profiles across all samples at each time point (5h, 20h, and 48hr post-infection). I stands for IAPV treatment, P stands for PBS sham control. The correlation coefficients are listed.

Supple Figure 11 motifs: Motifs of 6-mers in the differentially methylated regions after 5h, 20h, and 48h viral infections.

Supple Table 1 seq coverage: The sample information and sequencing coverage of each sample.

Supple Table 2_DEG 5h 20h 48h: DEG list after IPAV 5h, 20h, and 48h infection.

Supple Table 3 comparison to other genome papers and hypogeo tests 2: Gene list when compared to other genome papers.

Supple Table 4 hypergeo test raw data: Raw data of hypergeometric tests when compared to other genome papers.

Supple Table 5 Overlap with Dm immune genes: Overlapping gene list comparing with immune genes in *Drosophila* post viral infections.

Supple Table 6 differentially_splicing_5_20_48h: Number of significant hits for differential transcript usage.

Supple Table 7 IAPV DMR 10p list with gene anno: DMR list with more than 10 percentage changes.

Supple Table 8_5h DMR 48h DEG with diff alt splicing: DMR 5h infection_DEG 48h infection list with different spliced genes. ). Tss (Transcription start site), Tts (Transcription termination site), ORF (Open Reading Frame), UTR (Untranslated Region), NMD (Nonsense-mediated decay), IDR (intrinsically disordered regions). Description of isoform changes categories are: Tss: Change in transcription start site; Tts: Change in transcription termination site; Last exon: Last exon changed; Intron Retention: Difference in intron retention; Intron structure: Different exon-exon junctions used; Exon number: Different number of exons; ORF seq similarity: Jaccard similarity of AA sequences below 0.9; ORF genomic: Change in genomic position of ORF; 5 utr length: Difference in length of 5’ UTR; 3 utr length: Difference in length of 3’ UTR; NMD status: Change in sensitivity to nonsense-mediated decay; Coding potential: Change in coding potential probability, above or below 0.7; Domains identified: Change in which protein domains were identified; Domain length: Change in length of overlapping domains; IDR identified: Difference in presence of intrinsically disordered regions; IDR length: Difference in length of intrinsically disordered regions; IDR type: Difference in presence of binding site in IDR; Signal peptide identified: Change in presence of signal peptide.
